# NEMO‐Binding Domain/IKKγ Inhibitory Peptide Alleviates Neuronal Pyroptosis in Spinal Cord Injury by Inhibiting ASMase‐Induced Lysosome Membrane Permeabilization

**DOI:** 10.1002/advs.202405759

**Published:** 2024-09-03

**Authors:** Yibo Geng, Junsheng Lou, Junnan Wu, Zhichao Tao, Ningning Yang, Jiaxuan Kuang, Yuzhe Wu, Jiacheng Zhang, Linyi Xiang, Jingwei Shi, Yuepiao Cai, Xiangyang Wang, Jiaoxiang Chen, Jian Xiao, Kailiang Zhou

**Affiliations:** ^1^ Department of Orthopaedics The Second Affiliated Hospital and Yuying Children's Hospital of Wenzhou Medical University Wenzhou 325027 China; ^2^ Zhejiang Provincial Key Laboratory of Orthopaedics Wenzhou 325027 China; ^3^ Department of Orthopedic Surgery The First Affiliated Hospital Zhejiang University School of Medicine Hangzhou 310003 China; ^4^ Department of Pharmacy The Quzhou Affiliated Hospital of Wenzhou Medical University Quzhou People's Hospital Quzhou 324000 China; ^5^ Renji College of Wenzhou Medical University Wenzhou 325027 China; ^6^ Cixi Biomedical Research Institute Wenzhou Medical University Ningbo 315300 China; ^7^ Molecular Pharmacology Research Center School of Pharmaceutical Science Wenzhou Medical University Wenzhou 325027 China

**Keywords:** lysosomal membrane permeabilization, NBD peptide, NF‐κB/p38‐MAPK signalling pathway, pyroptosis, spinal cord injury

## Abstract

A short peptide termed NEMO‐binding domain (NBD) peptide has an inhibitory effect on nuclear factor kappa‐B (NF‐κB). Despite its efficacy in inhibiting inflammatory responses, the precise neuroprotective mechanisms of NBD peptide in spinal cord injury (SCI) remain unclear. This study aims to determine whether the pyroptosis‐related aspects involved in the neuroprotective effects of NBD peptide post‐SCI.Using RNA sequencing, the molecular mechanisms of NBD peptide in SCI are explored. The evaluation of functional recovery is performed using the Basso mouse scale, Nissl staining, footprint analysis, Masson's trichrome staining, and HE staining. Western blotting, enzyme‐linked immunosorbent assays, and immunofluorescence assays are used to examine pyroptosis, autophagy, lysosomal membrane permeabilization (LMP), acid sphingomyelinase (ASMase), and the NF‐κB/p38‐MAPK related signaling pathway.NBD peptide mitigated glial scar formation, reduced motor neuron death, and enhanced functional recovery in SCI mice. Additionally, NBD peptide inhibits pyroptosis, ameliorate LMP‐induced autophagy flux disorder in neuron post‐SCI. Mechanistically, NBD peptide alleviates LMP and subsequently enhances autophagy by inhibiting ASMase through the NF‐κB/p38‐MAPK/Elk‐1/Egr‐1 signaling cascade, thereby mitigating neuronal death. NBD peptide contributes to functional restoration by suppressing ASMase‐mediated LMP and autophagy depression, and inhibiting pyroptosis in neuron following SCI, which may have potential clinical application value.

## Graphical abstract

Spinal cord injury (SCI) is a serious and potentially fatal disease, but there is currently no effective treatment for SCI. The current study shows that NBD peptide contributes to functional restoration by suppressing ASMase‐mediated LMP and autophagy depression, and inhibiting pyroptosis in neuron following SCI. These findings indicate that NBD peptide may have potential clinical application value.

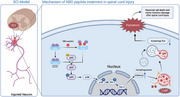

## 1 Introduction

Spinal cord injury (SCI), a potentially fatal disease with great severity, causes neurological dysfunction, leading to blockade or interruption of nerve signal transmission and resulting in motor, sensory, and autonomic dysfunction. Severe cases may lead to paralysis.^[^ ^1^, ^2^ ^]^ However, there is currently no highly effective treatment for SCI. SCI develops in distinct stages with characteristic features: primary injury and secondary injury. Secondary injury includes a cascade of responses such as oxidative stress, neuroinflammation, neuronal demise, and ischemia.^[^ ^3^, ^4^ ^]^ Given the unpredictability and irreversibility of primary injury, prevailing therapeutic endeavors are directed toward mitigating subsequent pathological alterations in secondary SCI to enhance clinical outcomes and prognosis for SCI patients.^[^ ^5^ ^]^ While the intricacies of secondary SCI remain incompletely understood, existing evidence suggests the prospect of interventions targeting neuroinflammation and cell death during this phase as promising strategies for SCI management.^[^ ^6^ ^]^

The intricate interaction between cell death and inflammation indicates a pivotal relationship.^[^ ^7^ ^]^ Pyroptosis, a newly identified mode of programmed cell death, occurs within diverse tissues, including the spinal cord and brain.^[^ ^8^, ^9^ ^]^ Pyroptosis involves the canonical and noncanonical pathways, and Caspase‐1 is involved in the former. Toll‐ and Nod‐like receptors recognize danger‐ or pathogen‐associated molecular patterns during the initiation phase of canonical pyroptosis, activating inflammasome‐related genes.^[^ ^10^ ^]^ This prompts the cleavage of pro‐Caspase‐1 to produce the active form. Active Caspase‐1 further processes pro‐IL‐1β and pro‐IL‐18 into their active forms, increasing the inflammatory response.^[^ ^11^ ^]^ Activated Caspase‐1 cleaves the Gasdermin‐D (GSDMD) protein to yield biologically active GSDMD‐N, which, in turn, mediates cell membrane dissolution, pyroptosis, and NLRP3 inflammasome activation.^[^ ^12^ ^]^ This culminates in the release of intracellular inflammatory factors, initiating a sequence of inflammatory events.^[^ ^13^ ^]^ Essentially, pyroptosis is a crucial pathological mechanism following SCI that represents an uncontrolled and dysregulated process in the subacute phase of injury.^[^ ^14^ ^]^ Moreover, neuronal pyroptosis is one of the main causes of neurological dysfunction and paralysis.^[^ ^15^ ^]^ Following SCI, the upregulation of NLRP3 inflammasome expression due to extensive signaling cascades results in neuronal pyroptosis, thereby exacerbating secondary injury.^[^ ^16^ ^]^ Therefore, the priority is to control the initiation and progression of pyroptosis in neurons to enhance the effectiveness of SCI treatment.

Autophagy is a lysosome‐dependent degradation process that uses lysosomes to break down damaged organelles, protein aggregates, and cytoplasmic proteins.^[^ ^17^ ^]^ This process is pivotal for preserving cellular homeostasis and ameliorating neurodegenerative diseases.^[^ ^18^ ^]^ Autophagy exerts neuroprotective effects against SCI by modulating microtubule dynamics and mediating axonal regeneration, thereby mitigating nerve damage.^[^ ^19^ ^]^ Notably, various investigations indicated the critical involvement of autophagy in neuronal processes.^[^ ^20^, ^21^ ^]^ Furthermore, the induction of autophagy has been shown to inhibit neuronal pyroptosis development.^[^ ^22^ ^]^ During autophagy, autophagosomes fuse with lysosomes to form autolysosomes.^[^ ^23^ ^]^ The material inside the autophagosomes is degraded by enzymes inside the lysosome, releasing useful molecules and energy for the cell to use. Consequently, lysosomes are integral participants in the autophagic process, and their functionality profoundly influences autophagy progression. Research indicated that an increase in lysosomal membrane permeability is associated with aging and various neurodegenerative disorders.^[^ ^24^, ^25^, ^26^ ^]^ When lysosomal membrane permeabilization (LMP) has occurred, enzymes and other molecules inside lysosomes leak into the cytoplasm and extracellular space, leading to cell death or impaired function and affecting the normal progression of autophagy.^[^ ^27^, ^28^, ^29^ ^]^ Therefore, maintaining the integrity and stability of lysosomal membranes is crucial for the normal progression of autophagy. Preventing LMP may restore autophagy flux in neurons and ultimately improve SCI. Acid sphingomyelinase (ASMase) is a crucial enzyme in sphingolipid metabolism and belongs to the sphingomyelinase family of enzymes.^[^ ^30^ ^]^ Interestingly, ASMase is mainly distributed on the inner side of lysosomal membranes, where it catalyzes the generation of ceramide (Cer) from sphingomyelin (SM).^[^ ^31^ ^]^ Cer is further degraded to sphingosine (Sph) by ASMase, which induces LMP.^[^ ^32^ ^]^ In addition, ASMase plays an important role in both the physiological and pathological processes of neurons, such as reducing neuronal death after traumatic brain injury by inhibiting ASMase.^[^ ^33^, ^34^ ^]^ Consequently, inhibiting ASMase to suppress LMP and restore autophagy flux is a viable strategy for enhancing neuronal survival and promoting functional recovery after SCI.

NEMO‐binding domain (NBD)/ IKKγ inhibitory peptide is a highly specific nuclear factor kappa‐B (NF‐κB) inhibitor.^[^ ^35^ ^]^ It inhibits TNF‐α‐induced NF‐κB activation by disrupting the interaction between the IKKγ/ NBD and IKKα and IKKβ.^[^ ^36^, ^37^ ^]^ Research has shown that NBD peptide exerts a therapeutic and neuroprotective effect by inhibiting inflammation in cerebral ischemia‒reperfusion injury.^[^ ^38^ ^]^ Moreover, our previous research demonstrated that peptide‐based medications exhibit notable therapeutic benefits in the treatment of SCI by preventing pyroptosis.^[^ ^15^, ^39^ ^]^ However, the role of NBD peptide in SCI treatment is still unclear. In addition, ischemia is a key pathological and physiological mechanism associated with SCI.^[^ ^40^ ^]^ Research findings indicated that the administration of the NBD peptide ameliorates neurological impairment caused by ischemia‒reperfusion injury, suggesting its potential use in the management of SCI.^[^ ^38^ ^]^ A recent study demonstrated that the NBD peptide inhibits the activity of ASMase.^[^ ^41^ ^]^ Moreover, research has shown that NF‐κB activates p38.^[^ ^42^ ^]^ Interestingly, it has been demonstrated that p38 activates ASMase through the Elk‐1/Egr‐1 pathway.^[^ ^43^ ^]^ However, whether the activity of ASMase in SCI is inhibited by NBD peptide via the NF‐κB/p38‐MAPK/Elk‐1/Egr‐1 pathway is still unknown. In this study, we used a mouse SCI model to examine the therapeutic efficacy of NBD peptides. Our primary objectives were to determine 1) the neuroprotective and motor recovery effects of NBD peptide in the context of SCI; 2) the involvement of autophagy, LMP, and pyroptosis in the underlying mechanism of NBD peptide treatment for SCI; 3) the potential role of ASMase in the NBD peptide‐mediated response to SCI; and (4) whether this response is mediated via the NF‐κB/p38‐MAPK pathway.

## 2 Results

### 2.1 NBD Peptide Promotes Post‐SCI Functional Recovery

**Figure 1:**
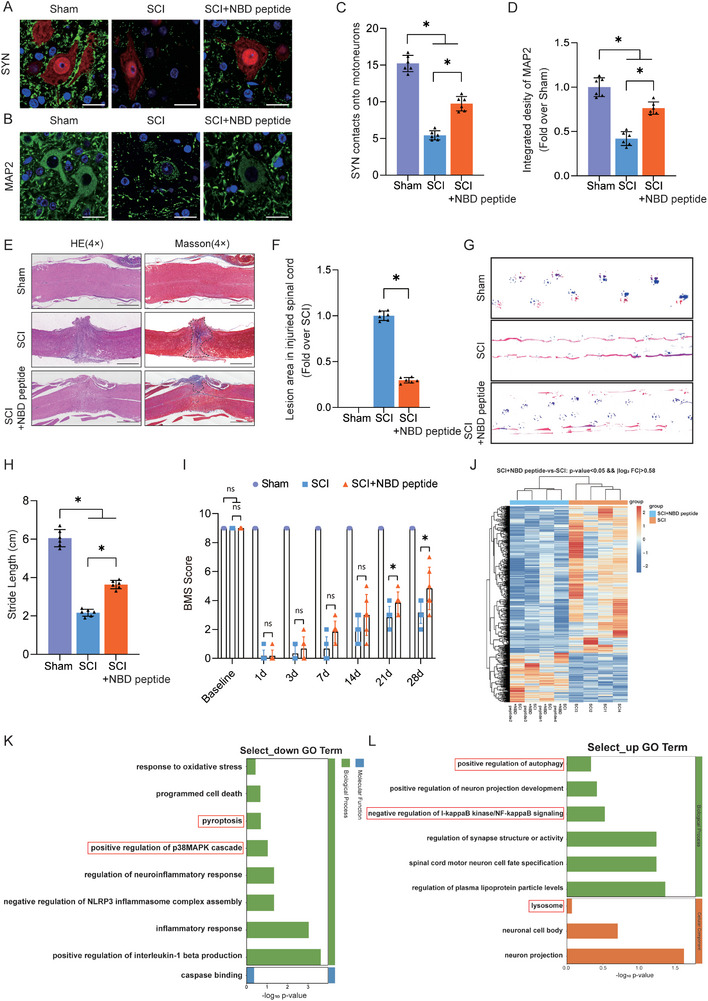
NBD peptide promotes post‐SCI functional recovery. A) Images of spinal cord cross‐sections from the corresponding groups were subjected to staining with SYN (green) and NeuN (red). (scale bar = 20 µm). B) Images of spinal cord cross‐sections from the corresponding groups were subjected to staining with MAP2 (green). (scale bar = 20 µm). C) Relevant quantitative results for motor neuron‐contacting synapse amounts on day 28 after SCI. D) MAP2 optical density within a spinal cord subjected to injury on day 28. E) Longitudinal spinal cord sections from the groups at 28 dpi were examined via HE and Masson staining (scale bar = 1000 µm). F) Quantitative investigations of Masson‐positive lesions within the spinal cords of the respective groups. G) On day 28 after SCI, images of mouse footprints were captured. Blue: fore paw print; Red: hind paw print. H) Stride length (cm) analyses of mice at 28 dpi. I) The groups' BMS scores were recorded on days 1, 3, 7, 14, 21, and 28 following SCI. J) Heatmap analysis of upregulated and downregulated genes induced by NBD peptide injection for 3 days into the mouse spinal cord (n = 4 mice per group). K) Perform GO enrichment analysis on the downregulated target genes to determine the biological processes affected by NBD peptide therapy. L) Perform GO enrichment analysis on the upregulated target genes to determine the biological processes affected by NBD peptide therapy. The data are presented as the means ± SEMs (n = 6 mice per group); ^*^ *p* < 0.05, indicates significant differences; ns, not significant. For the data in Figure 1C,D, F, H, I, statistical analysis was performed using two‐way ANOVA followed by Tukey's multiple comparison test. For the data in Figure 1J–L, statistical analysis was performed using an unpaired *t*‐test.

The effects of different doses of NBD peptide on motor function recovery in SCI mice were monitored to determine the optimal dose, and it was found that the optimal dose of NBD peptide was 500 µg kg^−1^ (Figure S1, Supporting Information). Furthermore, using a pharmacokinetic analysis, we measured the plasma concentration of NBD peptide at various times (0, 1, 2, 4, 8, 12, and 24 h) after the administration of a dose of 500 µg kg^−1^ and determined that the half‐life of the NBD peptide was 20.25 h (Figure S2, Supporting Information). Then, we systematically assessed the neuroprotective effects of NBD peptide on mice subjected to SCI by using comprehensive tissue staining and motor function evaluations. SYN, which is an indicator of motor cortical/spinal plasticity post‐injury, shows alterations in synaptic connectivity.^[^ ^44^ ^]^ The expression of MAP2 is associated with axonal regeneration and repair. When distal motoneurons exhibit abnormalities in the cytoskeleton, MAP2 levels decrease.^[^ ^45^ ^]^ At 28 days postinjury (dpi), immunofluorescence analysis showed a decrease in the number of anterior horn motor neurons, sparse distribution of MAP2, and a decrease in the number of SYN‐positive synapses on ventral motor neurons in the SCI group compared to the Sham group. Compared with untreated SCI mice, mice treated with NBD peptide showed an increase in the number of anterior horn motor neurons, a significant increase in the density of MAP2 distribution, and a significant increase in the number of SYN‐positive synapses on ventral motor neurons (**Figure** **1**A–D).

We performed HE and Masson staining at 3, 7, and 14 days after SCI to observe scar formation at different time points, as shown in Figure S3 (Supporting Information). We found that the glial scar was essentially stabilized 14 days after SCI, and a large area of the glial scar was observed at the injury site after SCI. Moreover, the area of the glial scar started to significantly shrink after day 7 following SCI with NBD peptide treatment. (Figure S3A–F, Supporting Information; Figure 1E–F). Notably, NBD peptide treatment mitigated the glial scar area, showing a significant therapeutic effect (Figure S3A–F, Supporting Information; Figure 1E–F). We performed a footprint analysis and assessed BMS scores to further explore the role of the NBD peptide in the restoration of locomotor function. The footprint analysis was performed at 28 dpi, and the results indicated a notable improvement in hind limb function in the SCI+NBD peptide group, while mice in the SCI group continued to exhibit an inability to lift their hind limbs (Figure 1G). Stride length data obtained from the footprint analysis revealed comparable patterns (Figure 1H). At 14, 21, and 28 dpi, SCI mice treated with NBD peptide exhibited significantly higher BMS scores than the SCI group (Figure 1I).

Transcriptome sequencing was performed on the SCI group and the SCI+NBD peptide group to determine the mechanism responsible for the observed effects of NBD peptide on SCI. Among 1026 differentially expressed genes (DEGs) (258 upregulated, 768 downregulated) between the SCI+NBD peptide and SCI groups (*p* < 0.05, Figure 1J), GO analysis indicated enrichment in processes related to autophagy, lysosomal activity, pyroptosis, and the NF‐κB and p38‐MAPK signaling pathway (Figure 1K,L). The transcriptome data indicated that pyroptosis, autophagy, and the NF‐κB and p38‐MAPK signaling pathways were involved in the therapeutic effect of the NBD peptide.

### 2.2 NBD Peptide Decreases Post‐SCI Pyroptosis in Neurons

**Figure 2:**
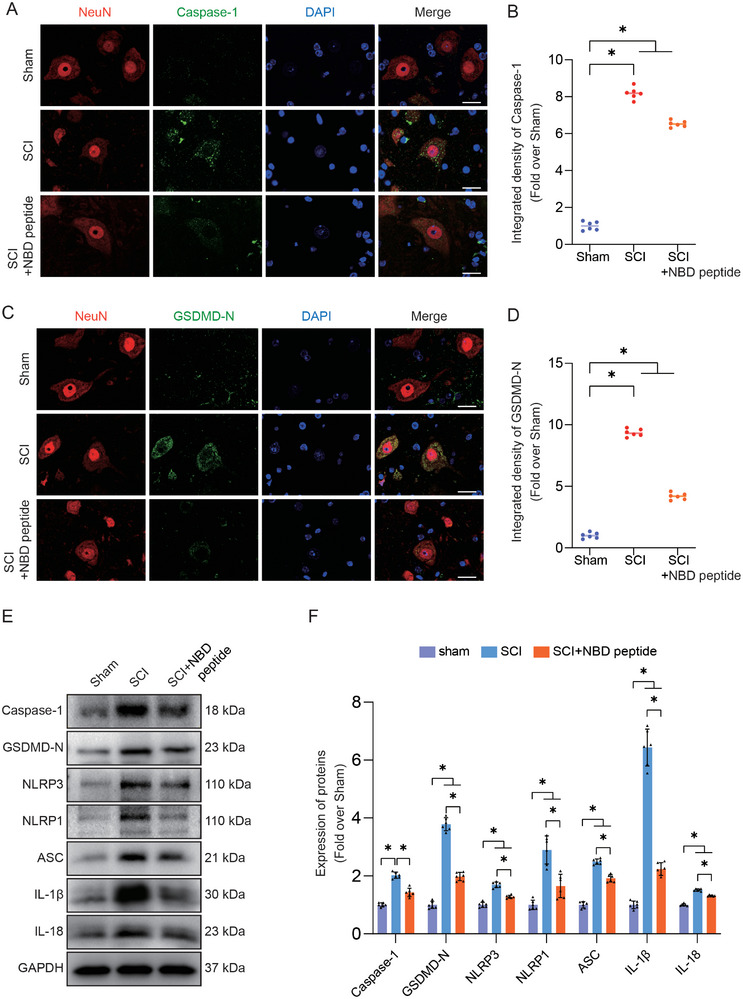
NBD peptide decreases post‐SCI pyroptosis in neurons. A) Caspase‐1 and NeuN were subjected to double‐IF staining in the spinal cords of the Sham, SCI, and SCI+NBD peptide groups (scale bar = 20 µm). B) The chart displays the relative strength of Caspase‐1 immunofluorescence staining in neurons within the specified groups. C) GSDMD‐N and NeuN were subjected to double‐IF staining in the spinal cords of the Sham, SCI, and SCI+NBD peptide groups (scale bar = 20 µm). D) The chart displays the relative strength of GSDMD‐N immunofluorescence staining in neurons within the specified groups. E) This figure depicts WB analysis of Caspase‐1, GSDMD‐N, NLRP3, NLRP1, ASC, IL‐1β, and IL‐18 expression levels in the Sham, SCI, and SCI+NBD peptide groups. GAPDH was utilized as a loading control. F) The optical density values for Caspase‐1, GSDMD‐N, NLRP3, NLRP1, ASC, IL‐1β, and IL‐18 were measured and evaluated in all groups. The data are presented as the means ± SEMs (n = 6 mice per group); ^*^ *p* < 0.05, indicates significant differences; ns, not significant. Statistical analysis was performed using two‐way ANOVA followed by Tukey's multiple comparison test.

Inflammation is an inevitable consequence of tissue injury caused by trauma. The prompt resolution of inflammation is a critical requirement to ensure efficacious host defense and proper cellular repair in the aftermath of trauma‐induced tissue injury. Excessive inflammation can lead to further cell death and worsen damage.^[^ ^46^ ^]^ Pyroptosis is triggered by inflammatory proteases that are dependent on cysteine and specific to aspartate.^[^ ^46^ ^]^ Pyroptosis has garnered significant interest due to its distinct role in the context of inflammation. To assess the potential inhibitory effects of NBD peptide on pyroptosis, we investigated key components of the pyroptotic pathway, including NLRP3, Caspase‐1, IL‐1β, NLRP1, ASC, IL‐18, and GSDMD‐N. IF staining in the SCI group demonstrated robust fluorescence signals for Caspase‐1 and GSDMD‐N in neurons. In contrast, the SCI+NBD peptide group exhibited only slight fluorescence signals (**Figure** **2**A–D). WB analysis was performed to assess the expression levels of Caspase‐1, IL‐1β, GSDMD‐N, NLRP1, ASC, IL‐18, and NLRP3. Pyroptosis‐related biomarkers showed notably higher expression in the SCI group than in the Sham group. Despite this, treatment with NBD peptide led to a discernible reduction in the expression of all seven pyroptosis‐related proteins compared to those in the SCI group (Figure 2E,F). The results indicated that NBD peptide effectively reduced neuronal pyroptosis following SCI.

The transcriptome sequencing analysis revealed that the oxidative stress response was inhibited following NBD peptide treatment in SCI (Figure 1J). Therefore, we investigated whether oxidative stress was inhibited in spinal cord tissue after NBD peptide treatment. Studies indicate that TAFA4 and its receptor FPR1 play crucial roles in the release of ROS.^[^ ^47^, ^48^, ^49^ ^]^ Therefore, we detected FPR1 and TAFA4 levels and used the ROS probe DHE to measure oxidative stress levels after SCI. As shown in Figure S4A,B (Supporting Information), enzymatic activity assays revealed increases in FPR1 and TAFA4 activities after SCI, which was subsequently decreased following the administration of NBD peptide. Furthermore, DHE staining also exhibited the same trend (Figure S4C,D, Supporting Information). These results indicated that NBD peptide effectively reduced oxidative stress levels following SCI.

### 2.3 NBD Peptide Potentiates Neuronal Autophagy Post‐SCI

**Figure 3:**
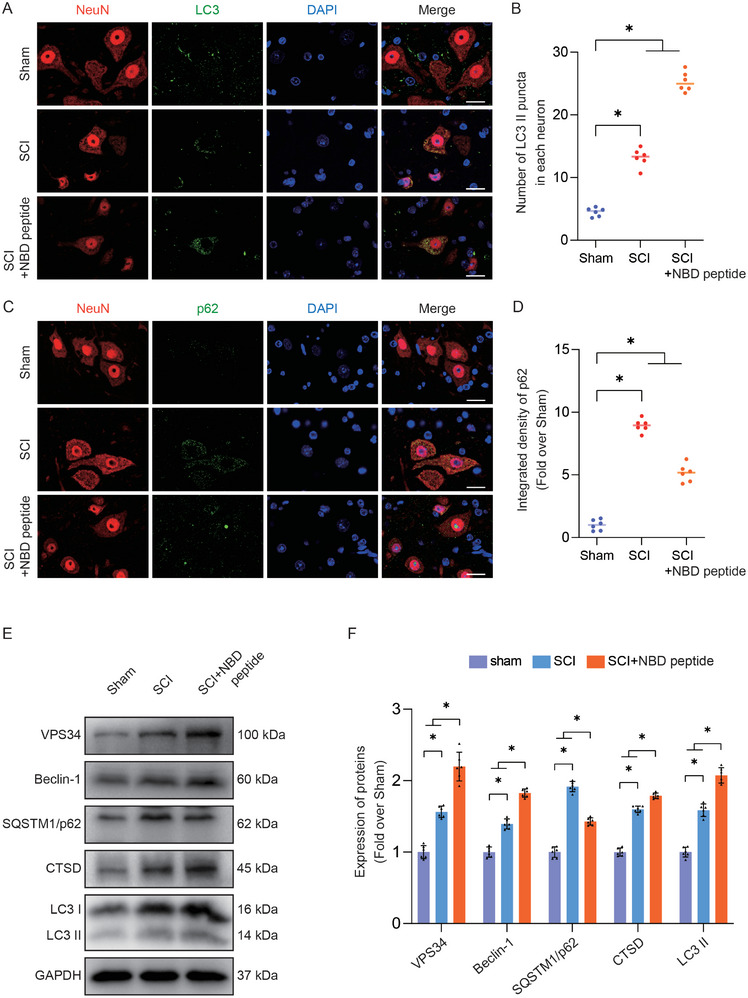
NBD peptide potentiates neuronal autophagy post‐SCI. A) LC3 and NeuN were subjected to double‐IF staining in the spinal cords of the Sham, SCI, and SCI+NBD peptide groups (scale bar = 20 µm). B) The chart displays the relative number of LC3 II puncta in neurons within the specified groups. C) SQSTM1/p62 and NeuN were subjected to double‐IF staining in the spinal cords of the Sham, SCI, and SCI+NBD peptide groups (scale bar = 20 µm). D) The chart displays the relative strength of SQSTM1/p62 immunofluorescence staining in neurons within the specified groups. E) This figure depicts WB analysis of VPS34, Beclin‐1, SQSTM1/p62, CTSD, and LC3II expression levels in the Sham, SCI, and SCI+NBD peptide groups. GAPDH was utilized as a loading control. F) The optical density values for VPS34, Beclin‐1, SQSTM1/p62, CTSD, and LC3II were measured and evaluated in all groups. The data are presented as the means ± SEMs (n = 6 mice per group); ^*^ *p* < 0.05, indicates significant differences; ns, not significant. Statistical analysis was performed using two‐way ANOVA followed by Tukey's multiple comparison test.

Elevated autophagic activity has been observed after SCI, and increasing evidence indicated that autophagy is a pro‐survival mechanism that intricately modulates neural cell death to confer neuroprotection.^[^ ^50^ ^]^ To explore the effect of NBD peptide on autophagic processes in neural cells following SCI, we examined autophagosomal protein expression (VPS34, Beclin‐1, LC3), the lysosomal enzyme CTSD, and SQSTM1/p62 at 3 days post‐SCI. As shown in **Figure** **3**A,B, the abundance of LC3 II puncta per neuron was increased post‐SCI and further increased following the administration of NBD peptide at 3 days post‐injury. Moreover, NBD peptide administration decreased SQSTM1/p62 in neurons in spinal cord lesions, in contrast to that in the SCI group (Figure 3C,D). Additionally, Western blot analysis revealed an increase in LC3 II, CTSD, VPS34, total LC3, and Beclin1 expression and a reduction in the expression of SQSTM1/p62 after NBD peptide treatment (Figure 3E,F). These observations showed that NBD peptide induces autophagic‐lysosomal activity, thereby enhancing autophagy in neurons following SCI.

### 2.4 Inhibiting Autophagy Abrogates NBD Peptide‐Mediated Neuronal Pyroptosis Inhibition and Functional Recovery Following SCI

**Figure 4:**
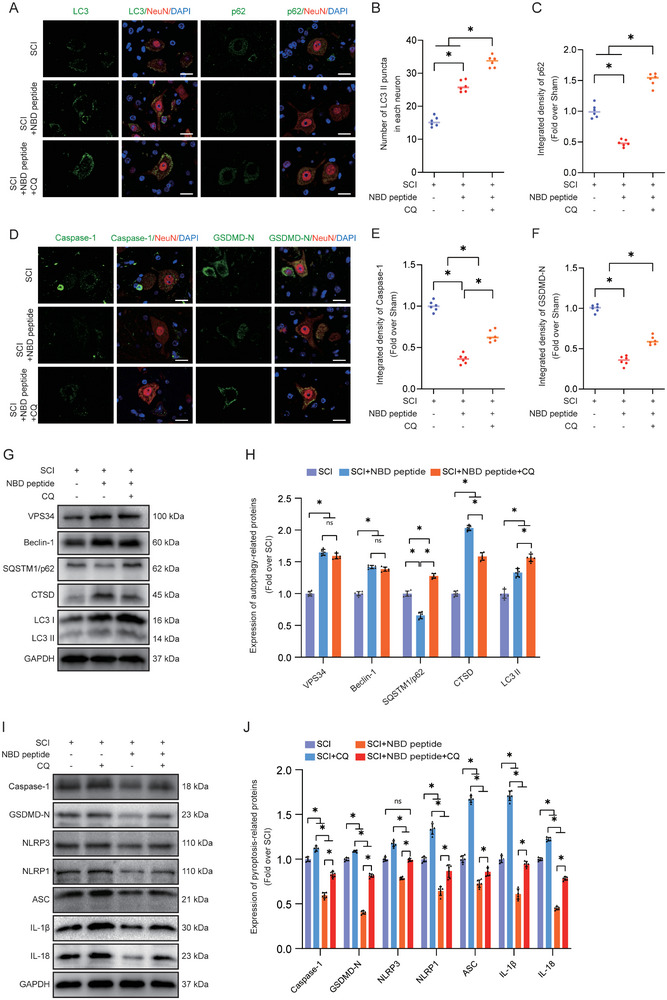
Inhibiting autophagy by CQ abrogates NBD peptide‐mediated neuronal pyroptosis inhibition following SCI. A–C) The figure displays representative double immunostaining images of LC3/NeuN and p62/NeuN in the injured spinal cord lesions from each group (the SCI, SCI+NBD peptide, SCI+NBD peptide+CQ groups) at 3 dpi (scale bar = 20 µm). The graph displays the LC3 II puncta count and the integrated density of SQSTM1/p62 for each individual neuron. D–F) The figure displays representative double immunostaining images of Caspase‐1/NeuN and GSDMD‐N/NeuN in the injured spinal cord lesions from each group (the SCI, SCI+NBD peptide, SCI+NBD peptide+CQ groups) at 3 dpi (scale bar = 20 µm). The graph displays the relative strength of Caspase‐1 and GSDMD‐N for each individual neuron. G) Typical images of WB analyses of VPS34, Beclin‐1, SQSTM1/p62, CTSD, and LC3II in the injured spinal cord lesions. GAPDH was utilized as a loading control. H) The quantified data of WB results for proteins related to autophagy. I) This figure depicts WB analysis of Caspase‐1, GSDMD‐N, NLRP3, NLRP1, ASC, IL‐1β, and IL‐18 expression levels in the SCI, SCI+CQ, SCI+NBD peptide, SCI+NBD peptide+CQ groups. GAPDH was utilized as a loading control. J) The quantified data of Western blot results for proteins related to pyroptosis. The data are presented as the means ± SEMs (n = 6 mice per group); ^*^ *p* < 0.05, indicates significant differences; ns, not significant. Statistical analysis was performed using two‐way ANOVA followed by Tukey's multiple comparison test.

To confirm that the beneficial effect of NBD peptide on SCI recovery is attributed to the increased neuronal autophagy activity resulting in reduced pyroptosis, we used CQ, a classic inhibitor of autophagy flux that impairs lysosomal acidification.^[^ ^51^ ^]^ Immunostaining analysis revealed that the SCI+NBD peptide+CQ group exhibited a higher level of LC3II expression than the SCI+NBD peptide group. Furthermore, IF analysis revealed that SQSTM1/p62 was increased in SCI+NBD peptide+CQ mice compared to SCI+NBD peptide mice (**Figure** **4**A–C). The results demonstrated that NBD peptide increased autophagy flux in SCI mice, and this effect was inhibited by CQ. In addition, WB was used to investigate the impact of CQ on the effects of NBD peptides on autophagy‐related proteins. The results indicated no notable difference between Beclin‐1 and VPS34 protein levels in the SCI+NBD peptide and SCI+NBD peptide+CQ groups (Figure 4G,H). This finding indicated that CQ did not alter autophagosome recruitment in SCI, regardless of the presence of NBD peptide. Pharmacological investigations were performed to assess the effect of CQ on inhibiting autophagy flux. Western blot analysis showed a slight increase in LC3‐II levels in the SCI+NBD peptide+CQ group compared to the SCI+NBD peptide group. In addition, the SCI+NBD peptide+CQ group exhibited a significantly higher level of SQSTM1/p62 than the SCI+NBD peptide group (Figure 4A,C). As mentioned previously, the WB and IF results for LC3 and SQSTM1/p62 were consistent. These results suggest that the increase in autophagy induced by the NBD peptide was prevented by CQ due to its ability to block autophagy flux. Autophagy regulates neuronal cell death and exerts neuroprotective effects against SCI, while pyroptosis is a distinct process used by cells to determine their fate.^[^ ^52^ ^]^ Consequently, we analyzed pyroptosis factor expression levels in the three groups and showed that the inhibitory effect of NBD peptide on pyroptosis was mitigated by CQ. The IF results indicated that the SCI+NBD peptide+CQ group had a higher number of Caspase‐1‐ and GSDMD‐N‐expressing neurons than the SCI+NBD peptide group (Figure 4D–F). Western blotting corroborated these findings, revealing increased levels of pyroptosis‐related proteins in the SCI+NBD peptide+CQ group (Figure 4I,J). The results suggested that CQ partially reverses the inhibitory effects of NBD peptide on pyroptosis in neurons following SCI.

Subsequently, we evaluated whether the functional recovery mediated by NBD peptide could be attenuated by CQ. At 28 days post‐SCI, we performed HE/Masson staining, immunofluorescence analysis, and motor function testing in the SCI, SCI+NBD peptide, and SCI+NBD peptide+CQ groups. IF staining showed that compared to the SCI+NBD peptide group, the SCI+NBD peptide+CQ group had a reduced number of anterior horn motor neurons, a decreased surface density of MAP2, and a decreased number of SYN‐positive synapses on ventral motor neurons (Figure S5A–C). In addition, the SCI+NBD peptide+CQ group exhibited an increased area of glial scarring in the injured spinal cord region compared to the SCI+NBD peptide group (Figure S5D,E). Subsequently, we performed footprint analysis and assessed the BMS score. On Day 28 after injury, the SCI+NBD peptide group exhibited notable recovery of hind leg movement, demonstrating coordinated crawling. However, the SCI+NBD peptide+CQ group continued to exhibit hind leg dragging (Figure S5F,G, Supporting Information). The SCI+NBD peptide+CQ group exhibited significantly lower BMS values at 14, 21, and 28 dpi than the SCI+NBD peptide group (Figure S5H, Supporting Information). These findings suggested that the increase in autophagy induced by NBD peptide may be the underlying mechanism responsible for the superior outcomes observed in response to NBD peptide treatment following SCI.

### 2.5 NBD Peptide Attenuates LMP and Inhibits the Activity of ASMase in Neurons After SCI

**Figure 5:**
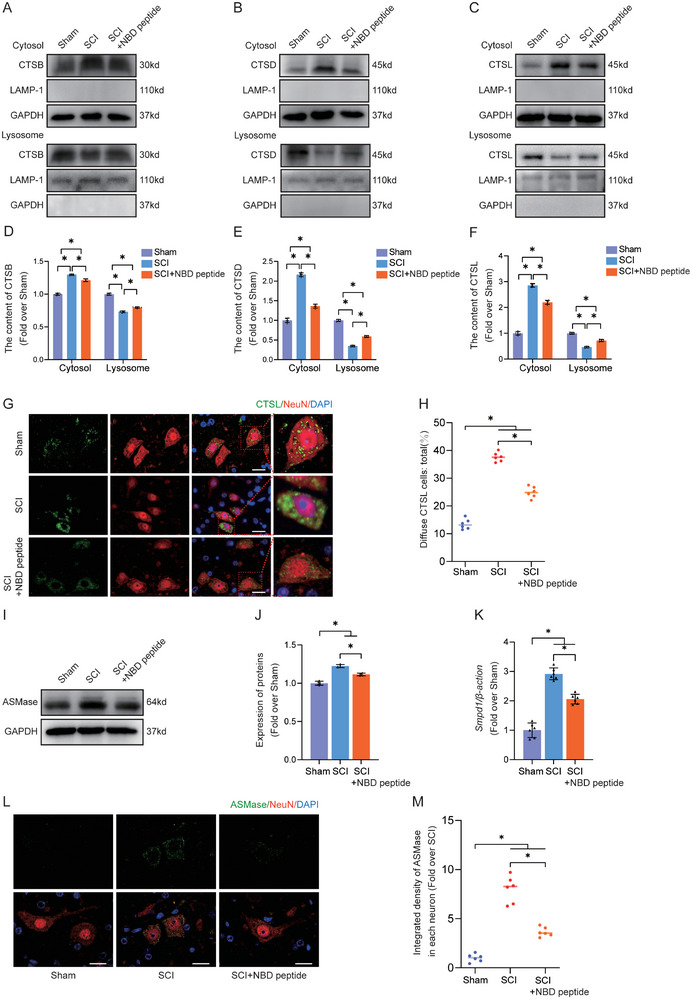
NBD peptide attenuates LMP and inhibits the activity of ASMase in neurons after SCI. A–C) The cytoplasmic and lysosomal protein levels of (A) CTSB, (B) CTSD, and (C) CTSL were measured in the spinal cords of the Sham, SCI, and SCI+NBD peptide groups. LAMP1 was utilized as a means of identifying the lysosomal fraction and as a loading control. GAPDH was used to identify the cytosolic fraction and as a loading control. D–F) Protein levels of (D) CTSB, (E) CTSD, and (F) CTSL were quantified in both cytoplasmic and lysosomal fractions extracted from the spinal cord. G) On postoperative day 3, IF staining was performed to detect the expression of NeuN and CTSL in the anterior horns of the spinal cords from the Sham, SCI, and SCI+NBD peptide groups (scale bar = 20 µm). H) A comparison was made between the three groups regarding the ratios of diffuse CTSL cells in the anterior horn of the spinal cord. I) ASMase protein levels in the spinal cords from the three groups on postoperative day 3. J) The optical density values for ASMase were measured and evaluated in all groups. K) The mRNA levels of ASMase gene in the injured spinal cord of the groups on day 3 after SCI. The data were normalized to β‐actin. L) On postoperative day 3, IF staining was performed to detect the expression of NeuN and ASMase in the anterior horns of the spinal cords from the Sham, SCI, and SCI+NBD peptide groups (scale bar = 20 µm). M) The quantitative integrated density of ASMase in each neuron is shown on the graph. The data are presented as the means ± SEMs (n = 6 mice per group); ^*^ *p* < 0.05, indicates significant differences; ns, not significant. Statistical analysis was performed using two‐way ANOVA followed by Tukey's multiple comparison test.

An earlier investigation demonstrated that the LMP pathways and consequent translocation of CTSB from lysosomes to the cytosol are signaling mechanisms that trigger pyroptosis.^[^ ^53^ ^]^ Moreover, there is growing evidence indicating that lysosomal impairment can hinder the proper flux of autophagy in neurological disorders, resulting in the accumulation of autophagosomes in neurons.^[^ ^54^, ^55^ ^]^ Given the profound effect of NBD peptide on pyroptosis suppression and autophagy flux in neurons in this study, we postulated that NBD peptide could affect lysosomal functionality in neurons after SCI. Lysosome‐enriched fractions were generated through spinal cord tissue subcellular fractionation, and the presence of lysosomal enzymes was validated by WB and IF analysis. WB analysis revealed increased lysosomal enzyme (including CTSL, CTSD, and CTSB) levels in cytosolic fractions in the SCI group compared to the Sham group, concomitant with decreased enzyme levels in lysosomal fractions (**Figure** **5**A–F). This was consistent with a decrease in enzyme concentrations in the lysosomal fractions. IF staining of CTSL and NeuN showed more diffuse CTSL distribution with fewer puncta in neurons in mice with SCI than in sham mice (Figure 5G,H). The findings indicated that the cytosol was infiltrated by lysosomal enzymes, providing evidence of LMP in neurons following SCI. Administration of supplementary NBD peptide to mice with SCI modified the distribution of lysosomal enzymes in neurons, indicating that NBD peptide mitigated neuronal LMP following SCI (Figure 5A–H).

There is a compromise in the functional integrity of lysosomal membranes. This compromise is a breach of the protective barrier, culminating in lysosomal content leakage into the cytoplasm. Phosphatides are widely recognized as essential constituents of neuronal bilayer membranes.^[^ ^56^ ^]^ ASMase can catalyze the conversion of sphingomyelin (SM) to ceramide (Cer).^[^ ^31^ ^]^ Cer is degraded by ASMase to sphingosine (Sph), thereby inducing LMP.^[^ ^32^ ^]^ We hypothesized that the protective effect of NBD peptide on the lysosomal membrane in SCI may be attributed to its ability to inhibit the ASMase enzyme. However, the role of ASMase in SCI has not been studied. To address this, we examined ASMase activity by Western blot analysis at 1, 3, 7, and 14 days post‐SCI (Figure S6A,B, Supporting Information). The results showed that the activity of ASMase peaked 3 days after SCI and gradually decreased thereafter. Moreover, we performed immunofluorescence experiments to examine the selective expression of ASMase in diverse cell types, including endothelial cells, microglia, neurons, and astrocytes (Figure S6C–J, Supporting Information). Notably, there is a significant increase in ASMase‐positive neurons, microglia, and astrocytes in the SCI group, with the highest increase observed in ASMase‐positive neurons (80.48%). Intriguingly, no significant difference was observed in the abundance of ASMase‐positive endothelial cells between the Sham and SCI groups. This result suggested that ASMase may play an important role in neurons. These findings shed light on the intricate dynamics of ASMase in SCI, opening avenues for a deeper understanding of its implications in neural pathophysiology.

We measured the activity levels of ASMase in neurons in the Sham, SCI, and SCI+NBD peptide groups. The results showed that ASMase levels were significantly upregulated after SCI, while NBD peptide inhibited ASMase activity (Figure 5I–K). The immunofluorescence results were similar: in the SCI group, the protein level of ASMase was higher than that in the Sham group, while the NBD peptide downregulated the protein level of ASMase after SCI (Figure 5L,M). These findings suggest that NBD peptide could suppress the activation of ASMase in neurons following SCI. Additionally, the enzymatic activity assay showed an increase in ASMase activity after SCI, which was subsequently reduced following the administration of NBD peptide (Figure S7A, Supporting Information). Subsequently, we performed spinal cord tissue subcellular fractionation and obtained cytosolic lysosome‐enriched fractions. Lysosomal fractions were assessed for CTSD and NAGLU by ELISA. Figure S7B,C (Supporting Information) shows that the lysosomal fractions in the SCI group exhibited decreased CTSD and NAGLU activity compared to the Sham group. Notably, the cytosolic fractions in the SCI group exhibited significantly higher levels of enzyme activity than those in the Sham group (Figure S7D,E, Supporting Information). The changes induced by SCI were notably mitigated by the administration of NBD peptide, indicating the potential restoration of lysosomal activity. The findings demonstrated that NBD peptide effectively suppressed the phosphorylation of ASMase and reduced the extent of lysosomal membrane impairment in neurons following SCI.

### 2.6 NBD Peptide Mitigates LMP, Increases Autophagy, and Suppresses Pyroptosis in Neurons by Inhibiting ASMase Post‐SCI

**Figure 6:**
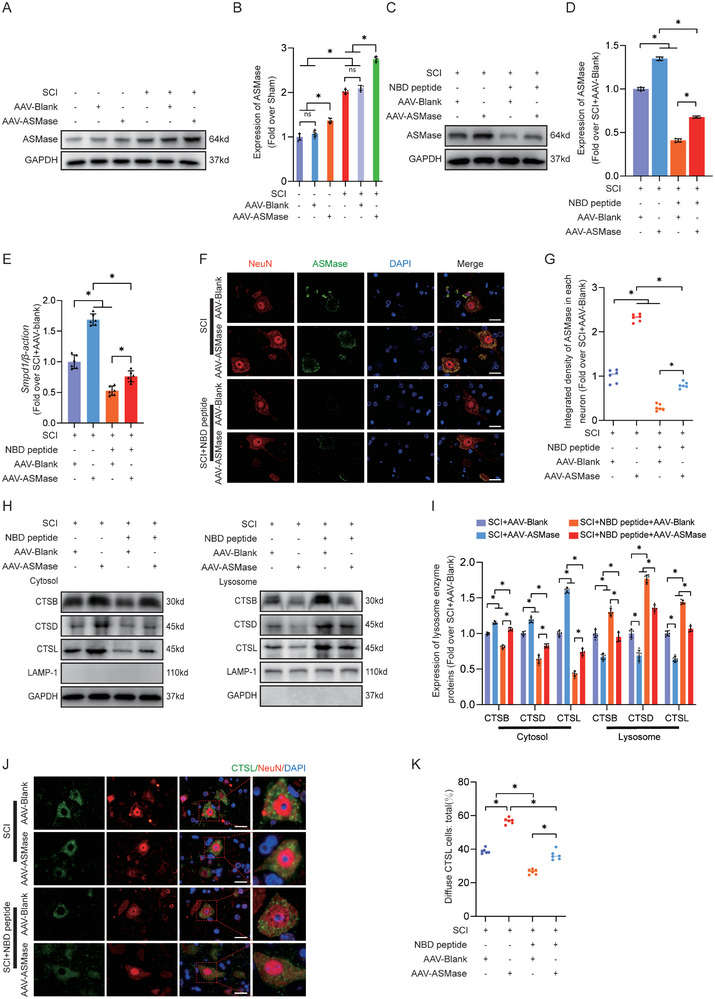
NBD peptide mitigates LMP and increases autophagy in neurons by inhibiting ASMase post‐SCI. A) This figure depicts WB analysis of ASMase expression levels in the Sham, Sham+AAV‐Blank, Sham+AAV‐ASMase, SCI, SCI+AAV‐Blank and SCI+AAV‐ASMase groups. GAPDH was utilized as a loading control. B) The expression levels of ASMase protein were measured in the damaged spinal cord. C) WB analysis of ASMase in the impaired spinal cords of the SCI+AAV‐Blank, SCI+AAV‐ASMase, SCI+NBD peptide+AAV‐Blank, and SCI+NBD peptide+AAV‐ASMase groups at 3 dpi. GAPDH was utilized as a loading control. D) The expression levels of ASMase protein were measured in the damaged spinal cord. E) The expression of ASMase gene in the injured spinal cord on day 3 after SCI was detected using qPCR. The data were normalized to β‐actin. F–G) Representative IF images of ASMase and NeuN in the impaired spinal cord areas from the SCI+AAV‐Blank, SCI+AAV‐ASMase, SCI+NBD peptide+AAV‐Blank, and SCI+NBD peptide+AAV‐ASMase groups at 3 dpi (scale bar = 20 µm). The data for quantification is presented on the right‐hand side. H) Protein concentrations of CTSB, CTSD, and CTSL were measured in the cytoplasm and lysosomes of the spinal cords from the four indicated groups for quantification. I) The protein levels of CTSB, CTSD, and CTSL were quantified in both cytoplasmic and lysosomal fractions obtained from the spinal cord. J) On postoperative day 3, immunofluorescence staining was performed to detect the expression of NeuN and CTSL in the anterior horns of spinal cords from four groups: SCI+AAV‐Blank, SCI+AAV‐ASMase, SCI+NBD peptide+AAV‐Blank, and SCI+NBD peptide+AAV‐ASMase (scale bar = 20 µm). (K) Comparison of the proportion of CTSL cells with diffuse staining in the anterior horn of the spinal cord across the four groups. The data are presented as the means ± SEMs (n = 6 mice per group); ^*^ *p* < 0.05, indicates significant differences; ns, not significant. Statistical analysis was performed using two‐way ANOVA followed by Tukey's multiple comparison test.

To examine the impact of NBD peptide on ASMase activation and its subsequent role in LMP, autophagy, and pyroptosis, we used AAV‐ASMase to instigate ASMase activity. In the rescue experiment, four distinct groups were examined: SCI+AAV‐Blank, SCI+AAV‐ASMase, SCI+NBD peptide+AAV‐Blank, and SCI+NBD peptide+AAV‐ASMase. WB, qPCR, and IF staining revealed a marked increase in ASMase levels in neurons in the SCI+AAV‐ASMase group compared with the SCI+AAV‐Blank group. Notably, the SCI+NBD peptide+AAV‐ASMase group consistently exhibited significantly increased ASMase expression levels in neurons compared to the SCI+NBD peptide+AAV‐Blank group (**Figure** **6**C–G). These results collectively showed substantial activation of ASMase following AAV‐ASMase transfection.

**Figure 7:**
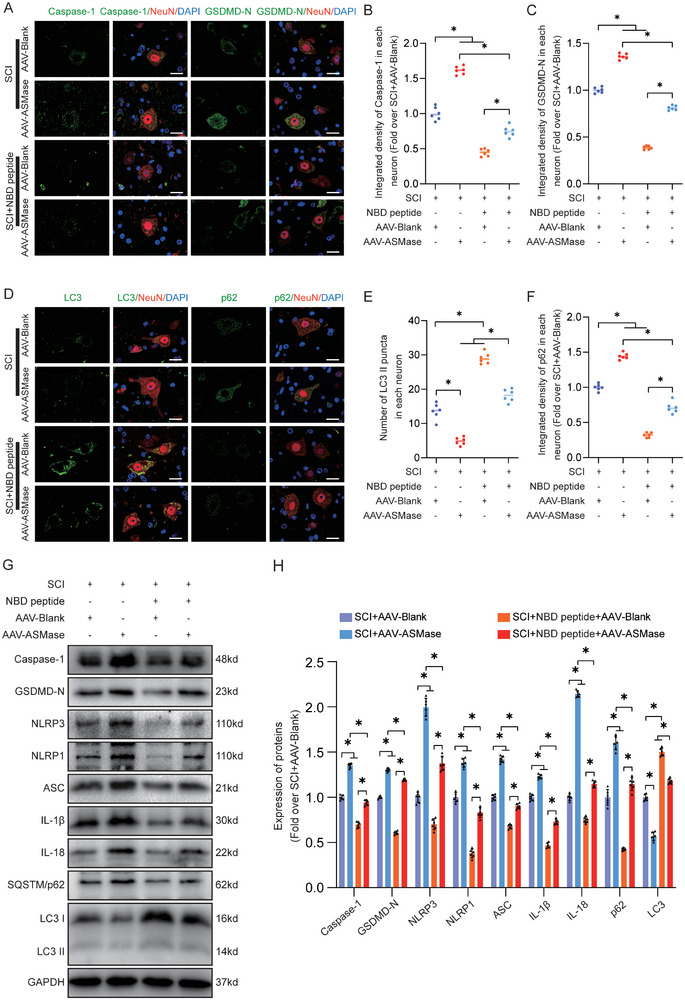
NBD peptide inhibits pyroptosis and enhances autophagy in neurons by downregulating ASMase. A–C) Double‐IF images were obtained to represent Caspase‐1/NeuN and GSDMD‐D/NeuN in spinal cord lesions from all four groups (SCI+AAV‐Blank, SCI+AAV‐ASMase, SCI+NBD peptide+AAV‐Blank, and SCI+NBD peptide+AAV‐ASMase) at 3 dpi (scale bar = 20 µm). The graph displays the integrated density of Caspase‐1 and GSDMD‐D in each neuron, measured quantitatively. D–F) Double‐IF images were obtained to represent LC3/NeuN and p62/NeuN in spinal cord lesions from all four groups (SCI+AAV‐Blank, SCI+AAV‐ASMase, SCI+NBD peptide+AAV‐Blank, and SCI+NBD peptide+AAV‐ASMase) at 3 dpi (scale bar = 20 µm). The graph displays the number of LC3 II puncta and the quantitative integrated density of SQSTM1/p62 in each neuron, measured quantitatively. G) The impaired spinal cord was subjected to WB analysis to obtain typical images of Caspase‐1, GSDMD‐N, NLRP3, NLRP1, ASC, IL‐1β, IL‐18, SQSTM1/p62, and LC3 II. GAPDH was utilized as a loading control. H) Quantification results of the related protein levels in the indicated groups. The data are presented as the means ± SEMs (n = 6 mice per group); ^*^ *p* < 0.05, indicates significant differences; ns, not significant. Statistical analysis was performed using two‐way ANOVA followed by Tukey's multiple comparison test.

The Western blot results shown in Figure 6H,I revealed a significant increase in lysosomal enzyme leakage into the cytoplasm in the SCI+NBD peptide+AAV‐ASMase group compared to the SCI+NBD peptide+AAV‐Blank group. The immunofluorescence data showed an increase in the level of diffuse CTSL staining in the cytoplasm of neurons in the SCI+NBD peptide+AAV‐ASMase group compared to the SCI+NBD peptide+AAV‐Blank group (Figure 6J,K). Additionally, the enzyme activity assay substantiated the increased ASMase levels in the SCI+AAV‐ASMase group relative to the SCI+AAV‐Blank group, a trend consistently observed in the SCI+NBD peptide+AAV‐ASMase group compared to the SCI+NBD peptide+AAV‐Blank group (Figure S8A, Supporting Information). ELISA analysis of lysosomal fractions revealed significantly decreased CTSD and NAGLU enzyme activities in the SCI+AAV‐ASMase group compared to the SCI+AAV‐Blank group. Similarly, the SCI+NBD peptide+AAV‐ASMase group exhibited lower CTSD and NAGLU enzyme activity in lysosomes than the SCI+NBD peptide+AAV‐Blank group (Figure S8B,C, Supporting Information). Conversely, the SCI+AAV‐ASMase group showed increased levels of CTSD and NAGLU enzyme activities in the cytoplasm compared to the SCI+AAV‐Blank group. In parallel, the SCI+NBD peptide+AAV‐ASMase group exhibited increased CTSD and NAGLU enzyme activities in the cytoplasm relative to the SCI+NBD peptide+AAV‐Blank group (Figure S8D,E, Supporting Information). Collectively, these findings indicated that the suppression of ASMase reduces lysosomal damage, resulting in the suppression of LMP in neurons. Due to the potent inhibitory effect of NBD peptide on ASMase, we posited its role in modulating pyroptosis and autophagy. The immunofluorescence results demonstrated a substantial decrease in the integrated density of GSDMD‐N and Caspase‐1 in neurons in the SCI+NBD peptide+AAV‐Blank group compared to the SCI+AAV‐Blank group. Intriguingly, this decrease was reversed in the SCI+NBD peptide+AAV‐ASMase group (**Figure** **7**A–C). Western blot analysis verified this effect, showing reduced levels of pyroptosis‐related proteins in the SCI+NBD peptide+AAV‐Blank group compared to the SCI+AAV‐Blank group, while the SCI+NBD peptide+AAV‐ASMase group exhibited increased protein levels (Figure 7G,H). As shown in Figure 7D,H, IF, and WB showed an increase in LC3II puncta in neurons and LC3II expression in the SCI+NBD peptide+AAV‐Blank group relative to the SCI+AAV‐Blank group. Strikingly, this upregulation was attenuated in the SCI+NBD peptide+AAV‐ASMase group. On the other hand, the SCI+NBD peptide+AAV‐ASMase group demonstrated an increase in SQSTM1/p62 protein levels in neurons. Compared to the SCI+ AAV‐Blank group, the SCI+NBD peptide+AAV‐Blank group exhibited a decrease in neuronal SQSTM1/p62 protein levels, as observed by IF and WB analyses. The SCI+NBD peptide+AAV‐ASMase group demonstrated an increase in SQSTM1/p62 protein levels in neurons (Figure 7D–H). In summary, our results indicated that NBD peptide effectively inhibits ASMase, leading to a reduction in LMP and the promotion of autophagy in mice with SCI. Next, the restoration of autophagy flux promotes functional recovery in mice with SCI by inhibiting pyroptosis.

Finally, we explored the therapeutic effects of NBD peptide on mice transfected with AAV‐ASMase to determine whether the amelioration in motor function post‐SCI was attributed to ASMase inhibition. As predicted, the SCI+NBD peptide+AAV‐Blank group showed a decrease in the area of glial scars, more ventral motor neurons, the upregulation of MAP2, and an increase in the number of SYN‐positive synapses onto ventral motor neurons compared with the SCI+NBD peptide+AAV‐ASMase group (Figure S9A–E, Supporting Information). Footprint analysis revealed reduced movement in the rear legs during crawling in the SCI+NBD peptide+AAV‐ASMase group compared to the SCI+NBD peptide+AAV‐Blank group (Figure S9F,G, Supporting Information). In addition, the SCI+NBD peptide+AAV‐ASMase group exhibited significantly lower BMS scores at 21 and 28 dpi than the SCI+NBD peptide+AAV‐Blank group (Figure S9H, Supporting Information). Furthermore, the enzyme activity assay confirmed increased FPR1 and TAFA4 activity levels in the SCI+AAV‐ASMase group compared to the SCI+AAV‐Blank group. This same pattern was noted in the comparison between the SCI+NBD peptide+AAV‐ASMase group and the SCI+NBD peptide+AAV‐Blank group (Figure S10A,B, Supporting Information). Furthermore, DHE staining also exhibited the same trend (Figure S10C,D, Supporting Information). These results indicate that NBD peptide inhibits oxidative stress after SCI by suppressing ASMase. In summary, our findings suggest that the therapeutic effect of NBD peptide is achieved by inhibiting ASMase, which in turn promotes the restoration of autophagy flux by inhibiting LMP to prevent pyroptosis.

### 2.7 NBD Peptide Inhibits ASMase Through the NF‐κB/p38‐MAPK/Elk‐1/Egr‐1 Signalling Pathway

**Figure 8:**
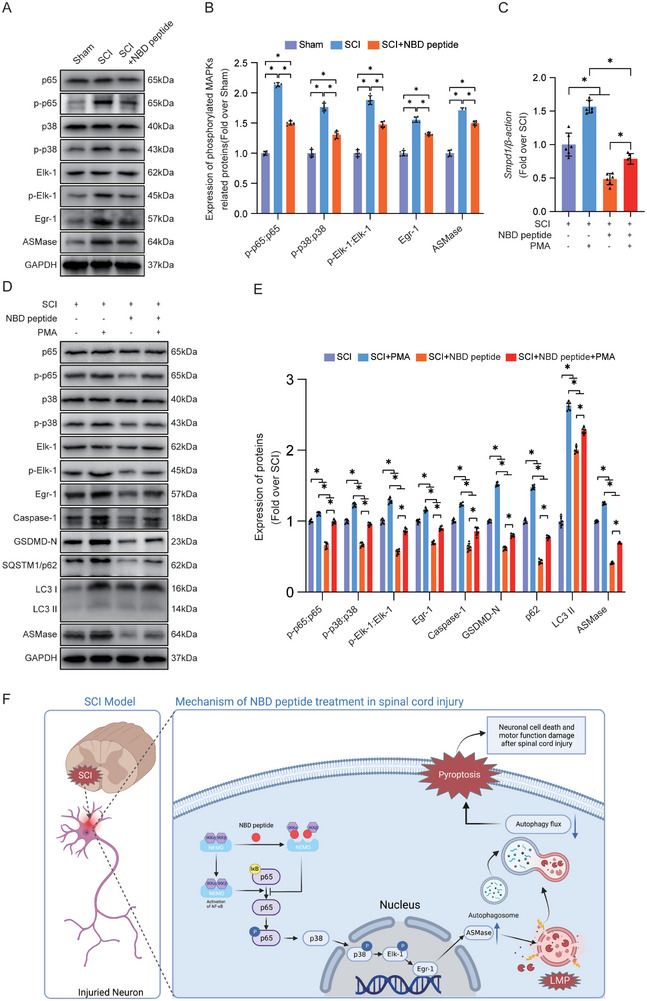
NBD peptide inhibits ASMase through the NF‐κB/p38‐MAPK/Elk‐1/Egr‐1 signaling pathway. A) The WB results of p65, p‐p65, p38, p‐p38, Elk‐1, p‐Elk‐1, Egr‐1, and ASMase expression in the injured spinal cord lesions in Sham, SCI, and SCI + SS‐31 groups are presented as typical images. GAPDH was utilized as a loading control. B) Quantification of the protein levels of p65, p‐p65, p38, p‐p38, Elk‐1, p‐Elk‐1, Egr‐1, and ASMase. C) The expression of ASMase gene in the injured spinal cord on day 3 after SCI was detected using qPCR. The data were normalized to β‐actin. D) The WB results of p65, p‐p65, p38, p‐p38, Elk‐1, p‐Elk‐1, Egr‐1, Caspase‐1, GSDMD‐N, SQSTM1/p62, LC3 and ASMase expression in the injured spinal cord lesions in SCI, SCI+PMA, SCI+NBD peptide and SCI+NBD peptide+PMA groups are presented as typical images. GAPDH was utilized as a loading control. E) Quantification of the protein levels of p65, p‐p65, p38, p‐p38, Elk‐1, p‐Elk‐1, Egr‐1, Caspase‐1, GSDMD‐N, SQSTM1/p62, LC3 and ASMase. F) Schematic diagram showing the potential protective effect of NBD peptide against SCI. The data are presented as the means ± SEMs (n = 6 mice per group); ^*^ *p* < 0.05, indicates significant differences; ns, not significant. Statistical analysis was performed using two‐way ANOVA followed by Tukey's multiple comparison test.

Transcriptome sequencing analysis revealed that NF‑κB and p38‐MAPK cascade was inhibited following NBD peptide treatment in SCI (Figure 1J). Therefore, our team investigated whether the NF‐κB/p38‐MAPK/*Elk‐1/Egr‐1* pathway was suppressed in spinal cord tissue after NBD peptide treatment. As shown in **Figure** **8**A,B, phosphorylation of p65, p38, and Elk‐1 was significantly increased following SCI. Furthermore, the protein levels of Egr‐1 and ASMase were significantly increased. After the administration of the NBD peptide, a discernible decrease was noted in the ratio of p‐p65/p65, p‐p38/p38 and p‐Elk‐1/Elk‐1, and there was a reduction in the protein expression levels of Egr‐1 and ASMase. These outcomes strongly suggested that NBD peptide exerts inhibitory effects on the NF‐κB/p38‐MAPK/Elk‐1/Egr‐1 pathway.

This study sought to assess the impact of the NF‐κB/p38‐MAPK signaling pathway on ASMase in response to NBD peptide treatment in SCI. Thus, we examined the effect of PMA, a well‐established NF‐κB agonist,^[^ ^57^ ^]^ on the NF‐κB/p38‐MAPK‐related signaling pathway. The results showed that the SCI+NBD peptide+PMA group exhibited increases in the ratio of p‐p65/p65 and the expression levels of ASMase compared to those in the SCI+NBD peptide group, thus substantiating the potency of PMA (Figure 8C–E). The data shown in Figure 8C,D reveal that the SCI+NBD peptide+PMA group exhibited increases in LC3 II, GSDMD‐N, Caspase‐1, and SQSTM1/p62, compared to the SCI+NBD peptide group. This underscores the efficacy of PMA in counteracting autophagic flux restoration and pyroptosis suppression induced by NBD peptide in the SCI+NBD peptide group. To further investigate the effects of PMA and NBD peptide treatment on ASMase and LMP, ELISA assays were performed. ELISA revealed a significant increase in the activity of ASMase in the SCI+NBD peptide+PMA group compared to the SCI+NBD peptide group (Figure S11A, Supporting Information). Additionally, ELISA demonstrated a noteworthy reduction in CTSD and NAGLU enzyme activity in lysosomes in the SCI+NBD peptide+PMA group compared to the SCI+NBD peptide group (Figure S11B,C, Supporting Information). Moreover, cytosolic fractions obtained from the SCI+NBD peptide+PMA group exhibited significantly increased levels of CTSD and NAGLU enzyme activity compared to the SCI+NBD peptide group (Figure S11D,E, Supporting Information). The results of ELISA demonstrated that PMA inhibited the inhibitory effect of NBD peptide on ASMase and LMP. Furthermore, we further investigated the relationship between the NF‐κB/p38‐MAPK/Elk‐1/Egr‐1 signaling pathway and oxidative stress by applying PMA. ELISA results showed that compared to the SCI+NBD peptide group, the activities of FPR1 and TAFA4 in the SCI+NBD peptide+PMA group were significantly increased (Figure S12A,B, Supporting Information). Furthermore, DHE staining also exhibited the same trend (Figure S12C,D, Supporting Information). These results indicated that NBD peptide inhibited oxidative stress after SCI through the NF‐κB/p38‐MAPK/Elk‐1/Egr‐1 signaling pathway. In summary, our findings suggest that NBD peptide effectively inhibits the activation of ASMase in SCI by inhibiting the NF‐κB/p38‐MAPK/Elk‐1/Egr‐1 signaling pathway.

## 3 Discussion

The effects of traumatic SCI include irreversible nerve damage, presenting a profound challenge for therapeutic interventions.^[^ ^1^ ^]^ This condition develops through distinct phases, notably primary injury, and subsequent secondary injury. The secondary injury phase is a focal point of extensive investigation and manifests as a cascade of neuroinflammation and programmed cell death as pivotal mechanistic drivers. Therapeutic interventions targeting the attenuation of proinflammatory cell death, specifically pyroptosis, can foster nerve regeneration and have emerged as promising strategies with notable therapeutic efficacy.^[^ ^58^, ^59^ ^]^ Our team's GO analysis showed that the significantly differentially expressed genes were enriched in biological processes related to pyroptosis, NF‐κB, and p38‐MAPK pathways. Our investigation also revealed that NBD peptide alleviates LMP‐induced autophagy impairment, and subsequently inhibits pyroptosis in neurons following SCI. This multifaceted treatment culminated in functional recovery in a murine SCI model. Furthermore, our results suggested that the neuroprotective effects of NBD peptide stem from its inhibitory effects on ASMase‐mediated LMP in neurons, which was mediated by the NF‐κB/p38‐MAPK/Elk‐1/Egr‐1 signaling pathway.

NBD peptide has been shown to have significant therapeutic effects on pneumonia, cerebral ischemia‒reperfusion injury, diabetes, and other conditions.^[^ ^38^, ^41^, ^60^ ^]^ However, the neuroprotective effect of NBD peptide on traumatic neural injury models is not yet clear. Therefore, we administered NBD peptide via intraperitoneal injection to mice to treat SCI. The findings revealed a substantial reduction in glial scar size in injured spinal cord tissue following treatment with NBD peptide. Moreover, a notable increase was observed in synaptic numbers of ventral motor and SYN‐positive neurons. Footprint analysis and BMS scores also demonstrated remarkable functional enhancement. These results suggested the neuroprotective effects of NBD peptide, in which ameliorates motor function in mice experiencing traumatic SCI. Given concerns regarding bladder complications in male mice post‐SCI, we used a female SCI mouse model. This decision was prompted by the challenges associated with manual bladder emptying in male mice following SCI, which is a procedural approach that was substantiated in prior research.^[^ ^61^, ^62^ ^]^ However, it is essential to acknowledge sex‐based differences in acute neuroinflammation and neural recovery in SCI models.^[^ ^63^ ^]^ Existing studies highlight the potential of estrogen to mitigate SCI severity by inhibiting proinflammatory pathways, decreasing oxidative stress, and preventing cell death.^[^ ^64^ ^]^ Conversely, testosterone exerts protective effects on motoneuron and muscle morphology post‐SCI.^[^ ^65^ ^]^ However, the distinct therapeutic effects of NBD peptide on SCI in the presence of various sex hormones remain unclear. Consequently, future investigations focusing on the therapeutic effects of NBD peptide on SCI in male mice are imperative for comprehensive insights.

Pyroptosis, an emerging paradigm of regulated cell death, manifests as a highly orchestrated process that is intricately associated with inflammatory cascades.^[^ ^53^ ^]^ The execution of pyroptosis involves inflammasome activation, which induces the creation of membrane pores, membrane distension, eventual membrane rupture, and the subsequent release of intracellular contents.^[^ ^53^ ^]^ This distinctive form of programmed cell death, which is a proinflammatory process, modulates the dynamics of the NLRP3 inflammasome, inducing signaling pathways orchestrated by Caspase‐1, Caspase‐4/5/11, and GSDMD, thereby amplifying the inflammatory responses.^[^ ^53^ ^]^ Accumulating evidence indicated the pivotal role of TXNIP in inducing interactions with the NLRP3 inflammasome during inflammatory episodes, thus activating the TXNIP/NLRP3 pyroptosis axis.^[^ ^66^, ^67^ ^]^ Moreover, preventing excessive neuronal death can be achieved by inhibiting the aforementioned pathway. Our results indicated that NBD peptide significantly downregulated pyroptosis levels in neurons post‐SCI. Autophagy has been shown to selectively breakdown the NLRP3 inflammasome, resulting in the inhibition of pyroptosis.^[^ ^68^ ^]^ Studies demonstrated that autophagy flux is upregulated or downregulated in response to CNS damage, depending on the severity and location of the injury.^[^ ^50^ ^]^ Studies have shown that enhancing neuronal autophagy following CNS injury suppresses pyroptotic death in neurons.^[^ ^69^, ^70^, ^71^ ^]^ Our results also confirmed that when autophagy flux was blocked, pyroptosis was significantly increased in neurons after SCI. Moreover, our results showed that after treatment with NBD peptide, neuronal autophagy flux was activated and pyroptosis in neurons was greatly inhibited after SCI. In addition, the inhibition of autophagy by CQ partially counteracted the effect on neuronal pyroptosis and the subsequent functional recovery induced by NBD peptide treatment. According to our findings, NBD peptide may prevent pyroptosis through a separate and distinct mechanism. However, further investigation is needed to fully determine this potential mechanism. In summary, our findings indicated that NBD peptide mitigated neuronal pyroptosis by enhancing autophagy flux, which has a positive impact on ameliorating SCI.

Autophagy plays a crucial role in the formation of glial scars following SCI.^[^ ^72^ ^]^ Investigating the biological mechanisms by which autophagy regulates glial scar formation has become a focal point of current research. Previous studies have shown that neuroinflammation and oxidative stress are closely associated with the glial scar.^[^ ^73^, ^74^, ^75^ ^]^ When inflammation persists, astrocytes undergo changes in the neuroinflammatory environment, including proliferation and hypertrophy, resulting in the formation of the main components of the glial scar.^[^ ^73^ ^]^ Moreover, inflammatory mediators stimulate astrocytes to express more cell adhesion molecules and extracellular matrix (ECM) components, which further promote the formation of the glial scar.^[^ ^76^, ^77^ ^]^ Oxidative stress also stimulates astrocytes to express more cell adhesion molecules and ECM components, further promoting the formation of the glial scar.^[^ ^75^, ^78^ ^]^ In addition, the induction of autophagy in the injured spinal cord can effectively inhibit neuroinflammation and oxidative stress.^[^ ^79^, ^80^ ^]^ Our research indicates that the NBD peptide reduces LMP by inhibiting ASMase, thereby increasing autophagy activity. NBD peptide‐induced autophagy may suppress the promotion of glial scar formation by neuroinflammation and oxidative stress following SCI, which warrants further investigation.

LMP, which is a consequential manifestation of substantial lysosomal dysfunction, occurs in response to diverse cellular stresses, including ROS, intralysosomal Fenton reactions, and other perturbations.^[^ ^81^ ^]^ In recent years, the involvement of LMP in CNS injury has been the focal point of extensive research.^[^ ^27^, ^28^, ^82^ ^]^ The accumulation of neuronal autophagosomes and the inhibition of autophagy flux in neural disorders are direct outcomes of lysosomal impairment.^[^ ^54^, ^55^ ^]^ Notably, pharmaceutical interventions targeting SCI by inhibiting LMP remains limited. Based on our findings, we posited that NBD peptide may relieve autophagy impairment by modulating LMP. Furthermore, our results substantiate our hypothesis that NBD peptide may inhibit the transfer of lysosomal enzymes from lysosomes to the cytoplasm and reestablishes the enzymatic functionality of lysosomes. According to our results, it is demonstrated that NBD peptide is a mitigating force in SCI, exerting its effect by mitigating LMP, and subsequently enhancing autophagy flux in neurons.

To elucidate the mechanism underlying the therapeutic effects of NBD peptide on SCI, we examined potential upstream pathways related to LMP, autophagy, and pyroptosis. Lysosomal membranes contain a large number of phospholipids, including sphingomyelin, phosphatidylcholine, phosphatidylethanolamine, phosphatidylinositol, and phosphatidylserine, which play a crucial role in maintaining membrane barrier function and lysosomal integrity.^[^ ^83^ ^]^ In the body, phospholipase exists in three forms depending on the pH: ASMase, neutral phospholipase, and alkaline phospholipase. Among them, ASMase accounts for 90% of the total activity of phospholipase.^[^ ^84^ ^]^ ASMase is mainly distributed on the inner membrane of lysosomes and is a crucial enzyme in sphingolipid metabolism. It has the ability to facilitate the transformation of SM to Cer through catalysis.^[^ ^31^ ^]^ Cer is further degraded into Sph by ASMase, which induces LMP.^[^ ^32^ ^]^ When ASMase disrupts lysosomes, it will lead to lysosomal dysfunction, which in turn hinders the autophagic process and ultimately results in cell death. However, there have been no reports on the role of ASMase in SCI so far. Therefore, this study is the first to investigate the function of ASMase in SCI. Our study revealed that after SCI, ASMase activation in neurons was inhibited by NBD peptide. In addition, NBD peptide inhibits LMP by suppressing the activation of ASMase in neurons following SCI, followed by an increase in the expression of autophagy‐related biomarkers. Ultimately, the restoration of autophagy flux alleviated pyroptosis in neurons. Thus, our investigation provides evidence that NBD peptide mitigates LMP, subsequently promoting autophagic processes, and ultimately inhibits pyroptosis in neurons by inhibiting ASMase activation in SCI.

Subsequently, we performed further research to explore the mechanism by which NBD peptide modulates the activity of ASMase. In this study, KEGG enrichment analysis showed that the NF‐κB and p38‐MAPK signaling pathway was significantly correlated with NBD peptide. The NBD peptide was originally designed as a highly specific inhibitor of NF‐κB. Currently, research has demonstrated a notable upregulation of NF‐κB expression following SCI.^[^ ^85^ ^]^ In response to NF‐κB stimulation, TAK1 or ASK activate p38 MAPK via MKK4.^[^ ^42^ ^]^ Activated p38 MAPK translocates to the nucleus, leading to the phosphorylation and activation of the transcriptional activator Elk‐1. Subsequently, Elk‐1 forms a ternary complex with CBP and SRF, and they transactivate the Egr‐1 gene promoter together. As an important nuclear transcription factor, Egr‐1 regulates the transcription and translation of ASMase by binding to the EGR site on the Smpd1 promoter.^[^ ^86^, ^87^ ^]^ In this study, it was found that NBD peptide treatment inhibited the NF‐κB/p38‐MAPK/Elk‐1/Egr‐1 signaling pathway in SCI. Moreover, we also reported for the first time that NBD peptide inhibited ASMase activity in an SCI model via the NF‐κB/p38‐MAPK/Elk‐1/Egr‐1 pathway.

The NBD peptide has shown promising therapeutic effects on the animal model of SCI, indicating great potential for clinical applications. However, the clinical use of NBD peptides is still limited to some extent. 1) Prior to clinical implementation, a comprehensive assessment of the toxicological impact and associated side effects of NBD peptide is needed. 2) Individuals with SCI require prolonged care and intervention. However, the bioavailability of NBD peptide is low, and it needs to be administered by injection or intravenous infusion, which is inconvenient for long‐term use by patients. Moreover, the distinctive architecture and physiological attributes of the gastrointestinal tract contribute to the decreased bioavailability and shortened half‐lives of peptide pharmaceuticals after oral administration. Therefore, in the future, NBD peptide must be mixed with materials such as hydrogels to create more a effective delivery system that can be administered directly to the SCI site. Alternatively, specific targeting peptides, such as targeted axonal import peptides, can be added to the NBD peptide molecule to create synthetic peptides for the treatment of SCI. This synthetic peptide can be administered orally or through other non‐invasive routes, thereby improving patient convenience and treatment outcomes. Both of these approaches enhance the sustained release of the NBD peptide and improve its bioavailability, thereby extending its therapeutic efficacy. 3) To maximize the therapeutic effect of NBD peptide on autophagy, combination therapy should be considered to achieve synergistic effects. For example, NBD peptide can be combined with other drugs targeting autophagy pathways to further enhance therapeutic efficacy.

There are still some limitations in this study that require further investigation. 1) Our primary emphasis was on the immediate repercussions of NBD peptide administration. Autophagy activity is inhibited during the acute phase of SCI, and the effect peaks on the third day.^[^ ^88^ ^]^ Consequently, we chose to initiate daily administration of NBD peptide before SCI induction and continued until 3 dpi. However, future investigations should prolong the treatment time to further understand the dynamic alterations in NBD peptide‐induced autophagy activation following SCI, as well as its long‐term impact on functional recovery post‐SCI. 2) Neurons are the most affected cells in SCI, and their death and damage are among the main outcomes of SCI.^[^ ^89^ ^]^ Studying neurons aids in enhancing our understanding of the function and regulatory mechanisms of the nervous system, thereby providing a better foundation for treating neurological diseases. After SCI, axonal growth and circuit reorganization are determined by the autonomous mechanisms of neurons and the interactions between neurons, glial cells, immune cells, and other cells.^[^ ^90^ ^]^ Prior research has demonstrated that the inhibition of ASMase leads to a significant increase in the number of oligodendrocytes, thereby promoting myelin repair in cases of acute and chronic demyelination.^[^ ^91^ ^]^ Therefore, the role of NBD peptide in treating SCI in other cells is also worth exploring.

## 4 Conclusion

Our study suggested that NBD peptide suppresses neuronal ASMase expression in SCI by suppressing the NF‐κB/p38‐MAPK/Elk‐1/Egr‐1 signaling pathway. The reduction of ASMase restored neuronal autophagy flux by alleviating LMP. Subsequently, the restoration of autophagy flux alleviated pyroptosis in neurons. These effects of NBD peptide led to improved outcomes in cases of SCI. Figure 8F shows a flowchart of this research. Overall, these results offer novel preclinical insights into the therapeutic potential of NBD peptide in the context of SCI. Future studies will further advance the clinical application of NBD peptide as a treatment for patients with SCI.

## 5 Experimental Section

### Animals

Due to the comparatively shorter length of the urethra in female animals, the facilitation of artificial urination is more feasible to prevent urinary retention subsequent to SCI. Consequently, female animals have been frequently used in SCI experimental research.^[^ ^92^ ^]^ In this study, healthy adult female C57BL/6J mice aged 6–8 weeks with an average body weight of 20–25 g was obtained. The mice were provided by the Experimental Animal Center (registration number SCXK [ZJ] 2015‐0001) affiliated with the institute in Zhejiang, People's Republic of China. The mice were maintained under standard conditions, including a 21–25 °C temperature range, a light/dark cycle (12 h), and humidity levels of 50–60%. Additionally, the mice were granted unrestricted access to food and water throughout the experimental period.

### Reagents and Antibodies

NBD peptide acetate (HPLC ≥ 99.15%, Cat. no. TP1615L) was purchased from TargetMol Chemicals Inc. (Boston, USA). Pentobarbital sodium, Masson staining reagents, and HE staining reagents were provided by Solarbio Science & Technology in Beijing, China. Dihydroethidium (DHE, Cat. no. GC30025) and Phorbol 12‐myristate 13‐acetate (PMA, Cat. no. GN10444) were provided by GlpBio in Montclair, California, USA. Additionally, chloroquine (CQ, Cat. no. C6628) was provided by Sigma‒Aldrich in St. Louis, Missouri, USA. Genecast Biotechnology in Shanghai, China, engineered the ASMase AAV virus and AAV blank control virus (CMV) (serotype# 9, without the fluorescent reporter gene). The titers of AAV‐ASMase and AAV‐Blank were determined by quantitative PCR, resulting in concentrations of 2.93E + 13 vg and 3E + 13 vg mL^−1^, respectively. Thermo Fisher Scientific in Waltham, USA, provided the Lysosome Enrichment Kit for Tissue & Cultured Cells (Cat. no. 89839). Primary antibodies against Beclin‐1 (Cat. no. 3738), LC3B (Cat. no. 3868), NLRP3 (Cat. no. 15101), p38 (Cat. no. 8690t), and p‐p38 (Cat. no. 4511t) were obtained from Cell Signaling Technology in Beverly, Massachusetts, USA. The Proteintech Group in Chicago, Illinois, USA, provided antibodies against VPS34 (Cat. no. 12452‐1), CTSD (Cat. no. 21327‐1), Caspase‐1 (Cat. no. 22915‐1), and GAPDH (Cat. no. 104941). Abcam in Cambridge, UK, supplied goat anti‐mouse IgG H&L Alexa Fluor 594 (Cat. no. ab150116), Alexa Fluor 488 (Cat. no. ab150113), goat anti‐rabbit IgG H&L Alexa Fluor 488 (Cat. no. ab150077), Alexa Fluor 594 (Cat. no. ab150080), and ASC (Cat. no. ab180799), Egr‐1 (Cat. no. ab300449), p65 (Cat. no. ab32536), p‐p65 (Cat. no. ab76302), SYN (Cat. no. 32127), MAP2 (Cat. no. ab5392), mouse monoclonal antibodies against NeuN (Cat. no. ab104224), rabbit monoclonal antibodies against NeuN (Cat. no. ab177487), p62/SQSTM1 (Cat. no. ab240635), CTSB (Cat. no. ab214428), and LAMP1 (Cat. no. ab24170) antibodies. The CTSL antibody (Cat. no. AF1515) was provided by R&D Systems in Minnesota, USA. Elk‐1 (Cat. no. A0789), IL‐1β (Cat. no. A1112), and IL‐18 (Cat. no. A1115) antibodies were provided by ABclonal Technology in Cambridge, MA, USA. NLRP1 (Cat. no. DF13187), p‐Elk‐1 (Cat. no. AF3212), ASMase (Cat. no. DF13384), GSDMD‐N (Cat. no. DF12275) antibodies were obtained from Affinity Biosciences in Ohio, USA. Servicebio in Wuhan, China, supplied CD31 antibodies (Cat. no. GB12063). Santa Cruz Biotechnology in Dallas, Texas, USA, provided GFAP (Cat. no. sc‐166458) and Iba‐1 (Cat. no. sc‐32725) antibodies. DAPI solution was obtained from Beyotime Biotechnology in Jiangsu, China. The BCA kit (Cat. no. 23227) and NE‐PER nuclear and cytoplasm extraction reagents (Cat. no. 78835) were acquired from Thermo Fisher Scientific (Rockford, IL, USA). Bioswamp in Wuhan, China, provided the ASMase ELISA kit (Cat. no. MU32986), the alpha‐N‐acetylglucosaminidase (NAGLU) ELISA kit (Cat. no. MU30875), and the CTSD ELISA kit (Cat. no. MU30978).

### Animal Model of SCI

Prior to the surgical procedure, each subject was intraperitoneally anesthetized with a 1% (w/v) solution of pentobarbital sodium (50 mg kg^−1^). Meticulous normal laminectomy was performed at the T9‐T10 level, ensuring spinal cord dorsal surface exposure without compromising the integrity of the dura mater. Subsequently, a 5 g rod was released from a height of 3 cm onto the spinal cord using a spinal cord impactor (W.M. Keck+, USA) to induce the desired injury. Following injury induction, a layer‐by‐layer closure of the muscles and skin was performed using 4‐0 silk sutures. Mice in the Sham group underwent an equivalent laminectomy procedure at the same spinal level but without injury. Throughout the post‐anesthesia recovery phase, the mice were housed in a temperature‐regulated environment until their body temperature was restored to physiological levels. After SCI, manual abdominal pressure was applied three times per day to empty the bladder until urinary function was restored. Antibiotics (30 mg kg^−1^ gentamicin sulfate) were administered once per day for three consecutive days after surgery.

### AAV Vector Injection

Consistent with prior investigations, the initial fortnight post‐SCI was the determined timeframe for AAV injection.^[^ ^69^, ^93^ ^]^ Consequently, within the first 2 weeks prior to surgical intervention, the Sham+AAV‐ASMase group, the SCI+AAV‐ASMase group, and the SCI+AAV‐ASMase+NBD peptide group underwent intraspinal injections of 2 µL of viral vectors in PBS at a distance of 1.5 mm from the spinal cord dorsal surface at the T9–T10 level using a microinjector. In parallel, the Sham+AAV‐Blank group, the SCI+AAV‐Blank, and SCI+AAV‐Blank+NBD peptide groups received an equivalent volume of inert viral vectors. Following the injection, the needle was retained in situ for 1 min to prevent potential virus/vehicle leakage. After the injection, the exposed muscles and spinal cord skin were meticulously sutured. After the surgical procedure, the mice were placed in a heated environment to facilitate postoperative convalescence. No instances of hind limb paralysis or other motor impairments were observed in any mice postinjection.

### Groupings

With the exception of RNA sequencing (n = 4, where “n” indicates biological replicates) and NBD peptide half‐life assay (n = 3), all analyses had consistent group sizes (n = 6). C57BL/6J mice (n = 320) were randomly allocated into 11 groups: Sham (n = 30), SCI (n = 52), SCI + NBD peptide (n = 52), SCI + CQ (n = 6), SCI + NBD peptide + CQ (n = 24), SCI + PMA(n = 12), SCI + NBD peptide + PMA (n = 12), Sham + AAV‐Blank (n = 6), Sham + AAV‐ASMase (n = 6), SCI + AAV‐Blank (n = 30), SCI + AAV‐ASMase (n = 30), SCI + AAV‐Blank + NBD peptide (n = 30), and SCI + AAV‐ASMase + NBD peptide (n = 30). The SCI + NBD peptide group, SCI + NBD peptide+PMA group, SCI+NBD peptide+CQ group, SCI+AAV‐Blank+NBD peptide group, and SCI + AAV‐ASMase + NBD peptide group were intraperitoneally injected with NBD peptide (500 µg kg^−1^) 30 min before surgery and further injected for 3 days after surgery.^[^ ^60^ ^]^ In the SCI, SCI + AAV‐Blank, Sham, and SCI + AAV‐ASMase groups, volumetric injections were conducted. Prior to NBD peptide treatment, intraperitoneal administration of chloroquine (CQ, 60 mg kg^−1^) or Phorbol 12‐myristate 13‐acetate (PMA, 20 ng kg^−1^) was performed 30 min in advance, and this process was repeated for three consecutive days. The doses of CQ and PMA and treatment time were determined by prior investigations of central nervous system (CNS) injury.^[^ ^57^, ^94^ ^]^ Excessive pentobarbital sodium was used to euthanize the mice, and tissue samples were collected at 3 and 28 days after surgery for histological examination.

### Evaluation of Functional Behavior

The Basso Mouse Scale (BMS) evaluations were performed at intervals of 0, 1, 3, 7, 14, 21, and 28 days post‐SCI. The BMS scale, which ranges from 0 to 9, identifies complete paralysis at a score of 0 and full motor function at a score of 9.^[^ ^95^ ^]^ The experimental assessments were performed in an open‐field environment. At 28 days post‐surgery, footprint analysis was performed. Blue and red dyes were used to stain the forelimbs and hindlimbs of the mice, respectively.^[^ ^96^ ^]^ The resulting footprints were used to gauge the extent of toe dragging and the length of each stride. Toe dragging quantification involved computing the total length of back leg drag divided by the overall walking distance. Hind limb distances that were next to each other were measured to determine stride length. Two researchers evaluated each mouse without knowledge of the treatment method.

### Tissue Pretreatment for HE & Masson Staining

On the 28th postoperative day, the mice were euthanized under excessive anesthesia by perfusion with ice‐cold 100 mm PBS (pH 7.4) and 4% (w/v) PFA administered through the heart. The entire spinal cord segment (10 mm, epicenter situated in middle) was fixed in 4% (w/v) PFA for 24 h. Each specimen was embedded in paraffin blocks and longitudinally sectioned. To perform HE staining, 5‐µm sections were sliced using a microtome and then affixed to poly‐L‐lysine‐coated slides as previously described.^[^ ^97^ ^]^ For Masson staining, deparaffinized longitudinal sections were stained with a mordant solution containing 10% potassium dichromate and 10% trichloroacetic acid, followed by hematoxylin staining of nuclei. The slices were differentiated in ethanol and hydrochloric acid, transitioned to a blue hue with a mild ammonia solution, and stained using Masson's solution in accordance with previously described protocols.^[^ ^97^ ^]^ Then, images were captured using an Olympus light microscope (Tokyo, Japan).

### Western Blotting (WB)

On the third day post‐SCI, the mice were subjected to humane euthanasia. Subsequently, a 1 cm segment of the spinal cord was meticulously dissected and homogenized within RIPA lysis buffer (ice‐cold) supplemented with a protease inhibitor cocktail and phosphatase inhibitor cocktail III. Protein extraction from the spinal cord samples was achieved using specialized reagents for protein isolation. Protein levels were quantified by using BCA assays. Equal amounts of protein (60 µg) were separated and transferred onto PVDF membranes (Millipore) by 12% (w/v) gel electrophoresis. Subsequently, the membranes were blocked for 2 h in 5% (w/v) skim milk, followed by overnight incubation at 4 °C with primary antibodies (1:1000 dilution) targeting specific proteins including IL‐1β, GSDMD‐N, IL‐18, Beclin‐1, Caspase‐1, ASC, CTSL, CTSB, CTSD, NLRP1, NLRP3, SQSTM1/p62, LC3, VPS34, LAMP1, GAPDH, ASMase, Elk‐1, p‐Elk‐1, p38, p‐p38, and Egr‐1. Then, the membranes were treated with HRP‐conjugated IgG secondary antibodies at ambient temperature for 2 h. Band signals were visualized and analyzed using a Bio‐Rad ChemiDoc XRS+ Imaging System in conjunction with an ECL immunodetection instrument.

### Immunofluorescence (IF) Analysis

Transverse sections of the spinal cord were meticulously processed for IF staining according to established protocols^.[^ ^98^ ^]^ After dewaxing and rehydration, the samples were subjected to rigorous fixation and high‐pressure antigen retrieval. Subsequently, the sections were blocked for 30 min at 37 °C with a 5% bovine serum albumin solution in PBS. After being blocked, the sections were incubated overnight at 4 °C with primary antibodies (1:200 dilution) targeting specific proteins. The proteins included Caspase‐1, GSDMD‐N, SQSTM1/p62, MAP2, SYN, NeuN, LC3, ASMase, CTSL, CD31, GFAP, and Iba‐1. On the next day, the sections were incubated with secondary antibodies at 37 °C for 1 h, followed by DAPI staining. Fluorescence microscopy imaging was performed using state‐of‐the‐art equipment from Olympus, Japan, to capture high‐resolution images of transverse sections positioned 3 mm rostral to the lesion site. Five random areas in the anterior horn of each specimen were selected for image analysis. Quantitative assessments were performed using ImageJ software to calculate the integrated density of MAP2, Caspase‐1, GSDMD‐N, SQSTM1/p62, and ASMase in each neuron. The evaluation process included the random selection of five areas in the anterior horn of each specimen. To quantify LC3 II puncta within the size range of 0.2–10 µm, which is indicative of autophagosomes, the IF images of each neuron were analyzed by using ImageJ software.^[^ ^99^ ^]^ SYN‐positive synapses on motor neurons were examined in a double‐blind manner through rigorous manual analysis. Additionally, ASMase‐positive cells, including microglia, astrocytes, and endothelial cells, were identified in a double‐blind manner, and the number of positive cells was normalized to the total number of imaged cells.

### Dihydroethidium Staining

The spinal cord tissue was subjected to gradient dehydration in 15%, 20%, and 30% sucrose solutions. The dehydrated tissue was placed on clean filter paper to remove excess moisture, covered with OCT, and cooled until the OCT was completely solidified. Subsequently, the tissue was cut into 5‐micron‐thick frozen sections of spinal cord tissue. Frozen spinal cord sections were stained with DHE (Beyotime, S0063) according to the manufacturer's protocol. Fluorescence microscopy imaging was performed using state‐of‐the‐art equipment from Olympus, Japan, to capture high‐resolution images of transverse sections positioned 3 mm rostral to the lesion site. Five random areas in the anterior horn of each specimen were selected for image analysis. Quantitative assessments were performed using ImageJ software to calculate the integrated optical density of DHE.

### Subcellular Partitioning and Isolation of the lysosome‐Rich Fraction

Tissue fragments from the spinal cord (5 mm in length) were subjected to ice‐cold homogenization by a Dounce tissue grinder. The Lysosome Enrichment Kit for Tissue and Cultured Cells (Thermo Fisher Scientific, 89839) was used to extract lysosomal fractions from the tissue homogenates by differential centrifugation according to the instructions. The resultant supernatant fractions were carefully preserved as the cytosolic fractions.

### Enzyme‐Linked Immunosorbent Assay (ELISA)

The enzymatic activities of FPR1 (MSKBIO, KT23004), TAFA4 (MSKBIO, KT23003), ASMase (Bioswamp, MU32986), CTSD (Bioswamp, MU30978), and NAGLU (Bioswamp, MU30875) were assessed using ELISA kits in accordance with the manufacturer's protocol. FPR1, TAFA4, ASMase, CTSD, and NAGLU were quantified by measuring the OD of the samples at 550 nm using a microplate reader. To ensure accuracy, a correction wavelength of 450 nm was used during the quantification process.

### Quantitative PCR (qPCR)

Total RNA was extracted from the spinal cord using TRIzol reagent according to the manufacturer's instructions. Quantitative analyses were completed via a two‐step reaction procedure: reverse transcription (RT) and PCR. Every RT reaction comprised 0.5 µg of RNA, 2 µL of 5×*TransScript* All‐in‐one SuperMix for qPCR, and 0.5 µL of gDNA remover in an overall volume of 10 µL. The reaction was conducted using a GeneAmp PCR System 9700 (Applied Biological Systems, USA) at 42 °C for 15 min, followed by 85 °C for 5 s. The 10 µL RT reaction mixture was then diluted 10‐fold in nuclease‐free water and incubated at −20 °C. Real‐time PCR was conducted using a LightCycler 480 II Real‐time PCR System (Roche, Switzerland) with a 10 µL PCR mix that included 1 µL of cDNAs, 5 µL of 2 × *PerfectStart* Green qPCR SuperMix, 0.2 µL of forward primer, 0.2 µL of reverse primer and 3.6 µL of nuclease‐free water. The reaction process was incubated in a 384‐well optic plate (Roche) at 94 °C for 0.5 min, followed by 45 cycles at 94 °C for 5 s and 60 °C for 30 s. All specimens were analyzed three times. After the PCR cycles, melting curve analyses were performed to verify the specific generation of the anticipated PCR products. The following primer sequences were developed in the laboratory and synthesized by Shanghai Generay Biotechnology based on the mRNA sequences acquired from the NCBI database: Smpd1, 5ʹ‐*AGGGGAGGAGCTGTTTTGTG*‐3ʹ (forward), and 5ʹ‐*CATTGCCAGGCTCGTAGACA*‐3ʹ (reverse). Finally, the mRNA expression levels were normalized to those of *β‐actin* and computed via the 2^−ΔΔCt^ approach.

### RNA Sequencing and Functional Enrichment Analysis

Three days post‐SCI, tissue samples were obtained, and tRNA was isolated using TRIzol reagent according to the manufacturer's instructions. The RNA and concentration were determined by a NanoDrop 2000 spectrophotometer (Thermo Scientific, USA). RNA integrity was assessed using an Agilent 2100 bioanalyzer (Agilent Technology, USA). Library preparation was performed according to the manufacturer's guidelines using a TruSeq Stranded mRNA LT Sample Prep Kit (Illumina, San Diego, CA, USA). OE Biotech Co., Ltd. (Shanghai, China) performed the transcriptome sequencing and subsequent analyses. The Illumina HiSeq X Ten system was used to generate 125 bp/150 bp paired‐end reads, and library construction was performed prior to sequencing. Raw reads were processed with Trimmomatic to eliminate poly‐N and low‐quality reads. Clean reads were retained for subsequent analysis. The results were mapped to the murine genome (GRCm38.p6) by using HISAT2. Cufflinks generated FPKM values for each gene, while HTSeqcount provided read counts. Differential expression analysis was performed by the DESeq (2012) package for R, and significance was set at *P* < 0.05 and a fold change > 2 or < 0.5. Layer clustering analyses were used to identify the expression features of differentially expressed genes (DEGs) among the groups and samples. GO analysis of DEGs was performed by an R‐based hypergeometric distribution.

### Pharmacokinetic Analysis

NBD peptide was administered to each mouse via a tail vein injection at a dose of 500 µg kg^−1^. The animals were divided into seven groups (the 0, 1, 2, 4, 8, 12, and 24 h groups) based on the retention time of NBD peptide in the mice, with each group consisting of three mice. Blood samples were collected from the mice in anticoagulant‐containing tubes. Plasma samples were collected after centrifuging the blood samples at 3000 × g for 10 min.^[^ ^100^ ^]^ The plasma concentration of NBD peptide was subsequently determined via high‐performance liquid chromatography‐mass spectrometry (LC‐MS). Finally, the concentrations were quantified using an internal standard method by comparing the peak area ratio of the drug to the internal standard and calculating the drug concentration in the plasma from a standard curve. Based on the one‐compartment model theory, the blood drug concentration‐time curve for NBD peptide was plotted, and a curve equation was obtained. The half‐life of NBD peptide was subsequently calculated using this equation.

### Statistical Analysis

Statistical analyses were carried out using GraphPad Prism Software, version 8.0.1. The data are expressed as the means ± SEMs, and rigorous statistical analysis of the measured parameters was performed. A two‐tailed, unpaired t‐test was used to compare data between two independent groups in this study. When the data exhibited a normal distribution, differences among three or more groups were examined by two‐way analysis of variance (ANOVA) and Tukey's multiple comparisons test. Nonparametric Mann‒Whitney U tests were used for groups in which the data did not conform to a normal distribution. One‐way ANOVA with least significant difference (LSD) post hoc analysis for groups with equal variances or Dunnett's T3 for groups with unequal variances was used for evaluation of significant differences between the groups. Significance levels were as follows: ^*^ *P* < 0.05.

### Ethical Statement

All experiments involving animals were conducted according to ethical policies, and the procedures were approved by the Ethics Committee of Wenzhou Medical University, China (Approval no. wydw 2017‒0096).

## Conflict of Interest

The authors declare no conflict of interest.

## Author Contributions

Y.G., J.L., and J.W. contributed equally to this work. Y. G., J. L., and J. W. performed experiments and analyzed the data. Z. T., N. Y., J. K., Y. W., J. Z., L. X., and J. S. provided critical appraisal. Y. G. and J. L. wrote the manuscript. Y. C., X. W., J. C., J. X., and K. Z. revised the manuscript. X. W., J. C., J. X., and K. Z. conceived the original idea and designed the experiments for this study. All authors read and approved the final manuscript.

## Supporting information

Supporting Information

## Data Availability

The data that support the findings of this study are available from the corresponding author upon reasonable request.

## References

[1] J. H. Badhiwala , J. R. Wilson , M. G. Fehlings , Lancet Neurol. 2019, 18, 24.30497967

[2] J. W. McDonald , C. Sadowsky , The Lancet 2002, 359, 417. 10.1016/S0140-6736(02)07603-1 11844532

[3] C. S. Ahuja , A. R. Martin , M. Fehlings , F1000Res 2016, 5. 10.12688/f1000research.7586.1 PMC4890313 27303644

[4] P. D. Smith , F. Puskas , X. Meng , J. H. Lee , J. C. Cleveland , M. J. Weyant , D. A. Fullerton , T. B. Reece , Circulation 2012, 126, 080275. 10.1161/CIRCULATIONAHA.111.080275 22965970

[5] S. Carelli , T. Giallongo , F. Rey , M. Colli , D. Tosi , G. Bulfamante , A. M. Di Giulio , A. Gorio , Cells 2019, 8, 329. 30965679 10.3390/cells8040329 PMC6523261

[6] K. Mortezaee , N. Khanlarkhani , C. Beyer , A. Zendedel , J. Cell. Physiol. 2018, 233, 5160. 29150951 10.1002/jcp.26287

[7] N. Peltzer , H. Walczak , Trends Immunol. 2019, 40, 387. 31003931 10.1016/j.it.2019.03.006

[8] L. Vande Walle , M. Lamkanfi , Curr Biol. 2016, 26, R568. 27404251 10.1016/j.cub.2016.02.019

[9] X. Hu , H. Zhang , Q. Zhang , X. Yao , W. Ni , K. Zhou , J. Neuroinflamm. 2022, 19, 242. 10.1186/s12974-022-02602-y PMC9531511 36195926

[10] P. Menu , J. E. Vince , Clin. Exp. Immunol. 2011, 166, 1. 21762124 10.1111/j.1365-2249.2011.04440.x PMC3193914

[11] S. Kesavardhana , R. K. S. Malireddi , T. D. Kanneganti , Annu. Rev. Immunol. 2020, 38, 567. 32017655 10.1146/annurev-immunol-073119-095439 PMC7190443

[12] K. Tsuchiya , Microbiol. Immunol. 2020, 64, 252. 31912554 10.1111/1348-0421.12771

[13] C. Zeng , R. Wang , H. Tan , Int. J. Biol. Sci. 2019, 15, 1345. 31337966 10.7150/ijbs.33568 PMC6643148

[14] X. Li , Z. Yu , W. Zong , P. Chen , J. Li , M. Wang , F. Ding , M. Xie , W. Wang , X. Luo , J. Neuroinflamm. 2020, 17, 263. 10.1186/s12974-020-01942-x PMC7487532 32891159

[15] H. Zhang , Y. Chen , F. Li , C. Wu , W. Cai , H. Ye , H. Su , M. He , L. Yang , X. Wang , K. Zhou , W. Ni , J. Neuroinflamm. 2023, 20, 6. 10.1186/s12974-023-02690-4 PMC9825014 36609266

[16] P. Broz , V. M. Dixit , Nat. Rev. Immunol. 2016, 16, 407. 27291964 10.1038/nri.2016.58

[17] R. Singh , A. M. Cuervo , Cell Metab. 2011, 13, 495. 21531332 10.1016/j.cmet.2011.04.004 PMC3099265

[18] T. Shintani , D. J. Klionsky , Science 2004, 306, 990. 15528435 10.1126/science.1099993 PMC1705980

[19] M. He , Y. Ding , C. Chu , J. Tang , Q. Xiao , Z.‐G. Luo , Proc. Natl. Acad. Sci. USA 2016, 113, 11324. 27638205 10.1073/pnas.1611282113 PMC5056063

[20] Y. Liu , X. Xue , H. Zhang , X. Che , J. Luo , P. Wang , J. Xu , Z. Xing , L. Yuan , Y. Liu , X. Fu , D. Su , S. Sun , H. Zhang , C. Wu , J. Yang , Autophagy 2019, 15, 493. 30304977 10.1080/15548627.2018.1531196 PMC6351122

[21] A. Shao , Z. Wang , H. Wu , X. Dong , Y. Li , S. Tu , J. Tang , M. Zhao , J. Zhang , Y. Hong , Mol. Neurobiol. 2016, 53, 18. 25399954 10.1007/s12035-014-8986-0

[22] D. Moujalled , A. Strasser , J. R. Liddell , Cell Death Differ. 2021, 28, 2029. 34099897 10.1038/s41418-021-00814-y PMC8257776

[23] X. W. Zhang , X.‐X. Lv , J.‐C. Zhou , C.‐C. Jin , L.‐Y. Qiao , Z.‐W. Hu , Adv. Exp. Med. Biol. 2021, 1208, 131. 34260026 10.1007/978-981-16-2830-6_9

[24] R. Gómez‐Sintes , M. D. Ledesma , P. Boya , Ageing Res. Rev. 2016, 32, 150. 26947122 10.1016/j.arr.2016.02.009

[25] N. Rodríguez‐Muela , A. M. Hernández‐Pinto , A. Serrano‐Puebla , L. García‐Ledo , S. H. Latorre , E. J. de la Rosa , P. Boya , Cell Death Differ. 2015, 22, 476. 25501597 10.1038/cdd.2014.203 PMC4326579

[26] S. Aits , M. Jäättelä , J. Cell Sci. 2013, 126, 1905. 23720375 10.1242/jcs.091181

[27] Y. Li , J. W. Jones , H. M. C. Choi , C. Sarkar , M. A. Kane , E. Y. Koh , M. M. Lipinski , J. Wu , Cell Death Dis. 2019, 10, 531. 31296844 10.1038/s41419-019-1764-1 PMC6624263

[28] C. Sarkar , J. W. Jones , N. Hegdekar , J. A. Thayer , A. Kumar , A. I. Faden , M. A. Kane , M. M. Lipinski , Autophagy 2020, 16, 466. 31238788 10.1080/15548627.2019.1628538 PMC6999646

[29] C. Sarkar , Z. Zhao , S. Aungst , B. Sabirzhanov , A. I. Faden , M. M. Lipinski , Autophagy 2014, 10, 2208. 25484084 10.4161/15548627.2014.981787 PMC4502690

[30] B. Henry , R. Ziobro , K. A. Becker , R. Kolesnick , E. Gulbins , Sphingolipids: Basic Science and Drug Development, Handbook of Experimental Pharmacology, Springer, Berlin, Germany 2013, pp. 77‐88. 10.1007/978-3-7091-1368-4_4 23579450

[31] S. A. Morad , M. C. Cabot , Nat. Rev. Cancer 2013, 13, 51. 23235911 10.1038/nrc3398

[32] C. Garcia‐Ruiz , J. M. Mato , D. Vance , N. Kaplowitz , J. C. Fernández‐Checa , J. Hepatol. 2015, 62, 219. 25281863 10.1016/j.jhep.2014.09.023

[33] S. H. Lee , A. R. Kho , S. H. Lee , D. K. Hong , B. S. Kang , M. K. Park , C. J. Lee , H. W. Yang , S. Y. Woo , S. W. Park , D. Y. Kim , B. Y. Choi , S. W. Suh , Int. J. Mol. Sci. 2022, 23, 14749.36499076

[34] W.‐Y. Ong , D. R. Herr , T. Farooqui , E.‐A. Ling , A. A. Farooqui , Expert Opin. Ther. Targets 2015, 19, 1725. 26243307 10.1517/14728222.2015.1071794

[35] J. Zhao , L. Zhang , X. Mu , C. Doebelin , W. Nguyen , C. Wallace , D. P. Reay , S. J. McGowan , L. Corbo , P. R. Clemens , G. M. Wilson , S. C. Watkins , L. A. Solt , M. D. Cameron , J. Huard , L. J. Niedernhofer , T. M. Kamenecka , P. D. Robbins , PLoS Biol. 2018, 16, e2004663. 29889904 10.1371/journal.pbio.2004663 PMC6013238

[36] M. J. May , F. D'Acquisto , L. A. Madge , J. Glo?ckner , J. S. Pober , S. Ghosh , Science 2000, 289, 1550. 10968790 10.1126/science.289.5484.1550

[37] L. A. Madge , M. J. May , Methods Mol. Biol. 2009, 512, 209. 19347279 10.1007/978-1-60327-530-9_11

[38] A. Desai , N. Singh , R. Raghubir , Neurochem. Int. 2010, 57, 876. 20868715 10.1016/j.neuint.2010.09.006

[39] Y. Xu , Y. Geng , H. Wang , H. Zhang , J. Qi , F. Li , X. Hu , Y. Chen , H. Si , Y. Li , X. Wang , H. Xu , J. Kong , Y. Cai , A. Wu , W. Ni , J. Xiao , K. Zhou , Redox Biol. 2023, 64, 102767. 37290302 10.1016/j.redox.2023.102767 PMC10267601

[40] K. Tsivelekas , D. S. Evangelopoulos , D. Pallis , I. S. Benetos , S. A. Papadakis , J. Vlamis , S. G. Pneumaticos , Cureus 2022, 14, e25475. 35800787 10.7759/cureus.25475 PMC9246426

[41] P. von Bismarck , S. Winoto‐Morbach , M. Herzberg , U. Uhlig , S. Schütze , R. Lucius , M. F. Krause , Pulm. Pharmacol. Ther. 2012, 25, 228. 22469869 10.1016/j.pupt.2012.03.002

[42] C. Zhao , G. Sun , Y. Li , K. Kong , X. Li , T. Kan , F. Yang , L. Wang , X. Wang , J. Orthop. Translat. 2023, 39, 147. 37188001 10.1016/j.jot.2023.02.005 PMC10175709

[43] T. Zheng , B. Zhang , C. Chen , J. Ma , D. Meng , J. Huang , R. Hu , X. Liu , K. Otsu , A. C. Liu , H. Li , Z. Yin , G. Huang , Proc. Natl. Acad. Sci. USA 2018, 115, E12313. 30541887 10.1073/pnas.1814705115 PMC6310843

[44] P. Wang , R. Yin , S. Wang , T. Zhou , Y. Zhang , M. Xiao , H. Wang , G. Xu , Med. Sci. Monit. 2021, 27, e931601. 34304239 10.12659/MSM.931601 PMC8317583

[45] S. L. González , J. J. López‐Costa , F. Labombarda , M. C. G. Deniselle , R. Guennoun , M. Schumacher , A. F. De Nicola , Cell. Mol. Neurobiol. 2009, 29, 27. 18584320 10.1007/s10571-008-9291-0 PMC11506021

[46] N. Van Opdenbosch , M. Lamkanfi , Immunity 2019, 50, 1352. 31216460 10.1016/j.immuni.2019.05.020 PMC6611727

[47] S. Zhu , X. Hu , S. Bennett , Y. Mai , J. Xu , Front. Cell Dev. Biol. 2022, 10, 911414. 35712659 10.3389/fcell.2022.911414 PMC9194834

[48] Y. Su , S. Zong , C. Wei , F. Song , H. Feng , A. Qin , Z. Lian , F. Fu , S. Shao , F. Fang , T. Wu , J. Xu , Q. Liu , J. Zhao , J. Cell. Physiol. 2019, 234, 14259. 30656690 10.1002/jcp.28124 PMC13483101

[49] L. Zhang , G. Wang , X. Chen , X. Xue , Q. Guo , M. Liu , J. Zhao , Sci. Rep. 2017, 7, 206. 28303030 10.1038/s41598-017-00314-5 PMC5428260

[50] M. M. Lipinski , J. Wu , A. I. Faden , C. Sarkar , Antioxid. Redox Signal. 2015, 23, 565. 25808205 10.1089/ars.2015.6306 PMC4545370

[51] I. Sergin , T. D. Evans , X. Zhang , S. Bhattacharya , C. J. Stokes , E. Song , S. Ali , B. Dehestani , K. B. Holloway , P. S. Micevych , A. Javaheri , J. R. Crowley , A. Ballabio , J. D. Schilling , S. Epelman , C. C. Weihl , A. Diwan , D. Fan , M. A. Zayed , B. Razani , Nat. Commun. 2017, 8, 15750. 28589926 10.1038/ncomms15750 PMC5467270

[52] C. Zhang , Z. Hu , R. Hu , S. Pi , Z. Wei , C. Wang , F. Yang , C. Xing , G. Nie , G. Hu , J. Hazard. Mater. 2021, 416, 126138. 34492927 10.1016/j.jhazmat.2021.126138

[53] X. Hu , H. Chen , H. Xu , Y. Wu , C. Wu , C. Jia , Y. Li , S. Sheng , C. Xu , H. Xu , W. Ni , K. Zhou , Int. J. Biol. Sci. 2020, 16, 2042. 32549752 10.7150/ijbs.45467 PMC7294939

[54] V. Ferrari , R. Cristofani , M. E. Cicardi , B. Tedesco , V. Crippa , M. Chierichetti , E. Casarotto , M. Cozzi , F. Mina , M. Galbiati , M. Piccolella , S. Carra , T. Vaccari , A. Nalbandian , V. Kimonis , T. R. Fortuna , U. B. Pandey , M. C. Gagliani , K. Cortese , P. Rusmini , A. Poletti , Neuropathol. Appl. Neurobiol. 2022, 48, e12818. 35501124 10.1111/nan.12818 PMC10588520

[55] P. Rusmini , K. Cortese , V. Crippa , R. Cristofani , M. E. Cicardi , V. Ferrari , G. Vezzoli , B. Tedesco , M. Meroni , E. Messi , M. Piccolella , M. Galbiati , M. Garrè , E. Morelli , T. Vaccari , A. Poletti , Autophagy 2019, 15, 631. 30335591 10.1080/15548627.2018.1535292 PMC6526812

[56] S. Alashmali , C. Walchuk , C. Cadonic , B. C. Albensi , M. Aliani , M. Suh , Nutr. Neurosci. 2022, 25, 1594. 33641632 10.1080/1028415X.2021.1885229

[57] Z. Zhang , Z. Zhu , X. Zuo , X. Wang , C. Ju , Z. Liang , K. Li , J. Zhang , L. Luo , Y. Ma , Z. Song , X. Li , P. Li , H. Quan , P. Huang , Z. Yao , N. Yang , J. Zhou , Z. Kou , B. Chen , T. Ding , Z. Wang , X. Hu , CNS Neurosci. Ther. 2023, 29, 3995. 37475184 10.1111/cns.14325 PMC10651991

[58] S. Xu , J. Wang , J. Jiang , J. Song , W. Zhu , F. Zhang , M. Shao , H. Xu , X. Ma , F. Lyu , Cell Death Dis. 2020, 11, 693. 32826878 10.1038/s41419-020-02824-z PMC7443136

[59] S. Xu , J. Wang , J. Zhong , M. Shao , J. Jiang , J. Song , W. Zhu , F. Zhang , H. Xu , G. Xu , Y. Zhang , X. Ma , F. Lyu , Clin. Transl. Med. 2021, 11, e269. 33463071 10.1002/ctm2.269 PMC7774461

[60] M. Kassan , S.‐K. Choi , M. Galán , M. Trebak , S. Belmadani , K. Matrougui , Diabetes/Metab. Res. Rev. 2015, 31, 39. 24652705 10.1002/dmrr.2542 PMC4829069

[61] D. Pan , Y. Li , F. Yang , Z. Lv , S. Zhu , Y. Shao , Y. Huang , G. Ning , S. Feng , J. Neuroinflamm. 2021, 18, 172. 10.1186/s12974-021-02215-x PMC8353762 34372877

[62] H. Fu , Y. Zhao , D. Hu , S. Wang , T. Yu , L. Zhang , Cell Death Dis. 2020, 11, 528. 32661227 10.1038/s41419-020-2733-4 PMC7359318

[63] Y. Li , R. M. Ritzel , Z. Lei , T. Cao , J. He , A. I. Faden , J. Wu , Brain Behav. Immun. 2022, 101, 1. 34954073 10.1016/j.bbi.2021.12.017 PMC8885910

[64] E. Brotfain , S. E. Gruenbaum , M. Boyko , R. Kutz , A. Zlotnik , M. Klein , Curr. Neuropharmacol. 2016, 14, 641. 26955967 10.2174/1570159X14666160309123554 PMC4981744

[65] J. S. Byers , A. L. Huguenard , D. Kuruppu , N.‐K. Liu , X.‐M. Xu , D. R. Sengelaub , J. Comp. Neurol. 2012, 520, 2683. 22314886 10.1002/cne.23066 PMC3960947

[66] J. Muri , M. Kopf , Nat. Rev. Immunol. 2021, 21, 363. 33340021 10.1038/s41577-020-00478-8

[67] R. Zhou , A. Tardivel , B. Thorens , I. Choi , J. Tschopp , Nat. Immunol. 2010, 11, 136. 20023662 10.1038/ni.1831

[68] Q. Sun , J. Fan , T. R. Billiar , M. J. Scott , Mol. Med. 2017, 23, 188. 28741645 10.2119/molmed.2017.00077 PMC5588408

[69] Y. Xu , X. Hu , F. Li , H. Zhang , J. Lou , X. Wang , H. Wang , L. Yin , W. Ni , J. Kong , X. Wang , Y. Li , K. Zhou , H. Xu , Oxid. Med. Cell Longevity 2021, 2021, 8186877. 10.1155/2021/8186877 PMC8548157 34712387

[70] C. Wu , H. Chen , R. Zhuang , H. Zhang , Y. Wang , X. Hu , Y. Xu , J. Li , Y. Li , X. Wang , H. Xu , W. Ni , K. Zhou , Int. J. Biol. Sci. 2021, 17, 1138. 33867836 10.7150/ijbs.57825 PMC8040310

[71] C. Gao , Y. Yan , G. Chen , T. Wang , C. Luo , M. Zhang , X. Chen , L. Tao , ACS Chem. Neurosci. 2020, 11, 4231. 33170612 10.1021/acschemneuro.0c00517

[72] S. Zhu , M. Chen , L. Deng , J. Zhang , W. Ni , X. Wang , F. Yao , X. Li , H. Xu , J. Xu , J. Xiao , Stem Cells Transl. Med. 2020, 9, 603. 32027101 10.1002/sctm.19-0282 PMC7180297

[73] Q.‐M. Pang , S.‐Y. Chen , Q.‐J. Xu , S.‐P. Fu , Y.‐C. Yang , W.‐H. Zou , M. Zhang , J. Liu , W.‐H. Wan , J.‐C. Peng , T. Zhang , Front. Immunol. 2021, 12, 751021. 34925326 10.3389/fimmu.2021.751021 PMC8674561

[74] J. C. T. Chio , N. Punjani , N. Hejrati , M.‐M. Zavvarian , J. Hong , M. G. Fehlings , Antioxid. Redox. Signaling 2022, 37, 184. 10.1089/ars.2021.0120 34465134

[75] M. V. Sofroniew , Trends Neurosci. 2009, 32, 638. 19782411 10.1016/j.tins.2009.08.002 PMC2787735

[76] H. S. Kwon , S. H. Koh , Transl Neurodegener 2020, 9, 42. 33239064 10.1186/s40035-020-00221-2 PMC7689983

[77] Y. Xia , L. Ding , C. Zhang , Q. Xu , M. Shi , T. Gao , F.‐Q. Zhou , D. Y. B. Deng , Neurosci. Bull. 2024, 40, 421. 37864744 10.1007/s12264-023-01128-4 PMC11003951

[78] Y. Chen , C. Qin , J. Huang , X. Tang , C. Liu , K. Huang , J. Xu , G. Guo , A. Tong , L. Zhou , Cell Prolif. 2020, 53, e12781. 32035016 10.1111/cpr.12781 PMC7106951

[79] Y. Li , Z. Lei , R. M. Ritzel , J. He , H. Li , H. M. C. Choi , M. M. Lipinski , J. Wu , Theranostics 2022, 12, 5364. 35910787 10.7150/thno.72713 PMC9330534

[80] J. Lou , M. Jin , C. Zhou , Y. Fan , L. Ni , Y. Mao , H. Shen , J. Li , H. Zhang , C. Fu , X. Mao , Y. Chen , J. Zhong , K. Zhou , L. Wang , J. Wu , Free Radical Biol. Med. 2024, 212, 133. 38142951 10.1016/j.freeradbiomed.2023.12.020

[81] C. Papadopoulos , B. Kravic , H. Meyer , J. Mol. Biol. 2020, 432, 231. 31449799 10.1016/j.jmb.2019.08.010

[82] N.‐K. Liu , J. S. Byers , T. Lam , Q.‐B. Lu , D. R. Sengelaub , X.‐M. Xu , J. Neurotrauma 2021, 38, 1327. 25386720 10.1089/neu.2014.3690 PMC9208724

[83] J. Piret , A. Schanck , S. Delfosse , F. Van Bambeke , B. K. Kishore , P. M. Tulkens , M.‐P. Mingeot‐Leclercq , Chem. Phys. Lipids 2005, 133, 1. 15589222 10.1016/j.chemphyslip.2004.08.002

[84] A. Mohamud Yusuf , N. Hagemann , D. M. Hermann , Neurosignals 2019, 27, 32. 31778304 10.33594/000000184

[85] B. Xu , J. Wang , S. Liu , H. Liu , X. Zhang , J. Shi , L. Ji , J. Li , J. Musculoskelet. Neuronal Interact. 2021, 21, 542. 34854394 PMC8672413

[86] A. Mylona , F.‐X. Theillet , C. Foster , T. M. Cheng , F. Miralles , P. A. Bates , P. Selenko , R. Treisman , Science 2016, 354, 233. 27738173 10.1126/science.aad1872 PMC5321235

[87] N. Mizutani , Y. Omori , Y. Kawamoto , S. Sobue , M. Ichihara , M. Suzuki , M. Kyogashima , M. Nakamura , K. Tamiya‐Koizumi , Y. Nozawa , T. Murate , Biochem. Biophys. Res. Commun. 2016, 470, 851. 26809095 10.1016/j.bbrc.2016.01.134

[88] K. Zhou , Z. Zheng , Y. Li , W. Han , J. Zhang , Y. Mao , H. Chen , W. Zhang , M. Liu , L. Xie , H. Zhang , H. Xu , J. Xiao , Theranostics 2020, 10, 9280. 32802192 10.7150/thno.46566 PMC7415792

[89] D. L. Torre , J. C. Md , Spine 1981, 6, 315. 7025250

[90] T. M. O'Shea , J. E. Burda , M. V. Sofroniew , J. Clin. Invest. 2017, 127, 3259. 28737515 10.1172/JCI90608 PMC5669582

[91] M. Chami , R. Halmer , L. Schnoeder , K. Anne Becker , C. Meier , K. Fassbender , E. Gulbins , S. Walter , PLoS One 2017, 12, e0178622. 28582448 10.1371/journal.pone.0178622 PMC5459450

[92] E. Lilley , M. R. Andrews , E. J. Bradbury , H. Elliott , P. Hawkins , R. M. Ichiyama , J. Keeley , A. T. Michael‐Titus , L. D. F. Moon , S. Pluchino , J. Riddell , K. Ryder , P. K. Yip , Exp. Neurol. 2020, 328, 113273. 32142803 10.1016/j.expneurol.2020.113273

[93] E. Ziemlinska , S. Kügler , M. Schachner , I. Wewiór , J. Czarkowska‐Bauch , M. Skup , PLoS One 2014, 9, e88833. 24551172 10.1371/journal.pone.0088833 PMC3925164

[94] J. Hu , H. Han , P. Cao , W. Yu , C. Yang , Y. Gao , W. Yuan , Am. J. Transl. Res. 2017, 9, 4607. 29118921 PMC5666068

[95] Z. He , S. Zou , J. Yin , Z. Gao , Y. Liu , Y. Wu , H. He , Y. Zhou , Q. Wang , J. Li , F. Wu , H. Z. Xu , X. Jia , J. Xiao , Sci Rep 2017, 7, 7661. 28794417 10.1038/s41598-017-08052-4 PMC5550423

[96] D. Jiang , F. Gong , X. Ge , C. Lv , C. Huang , S. Feng , Z. Zhou , Y. Rong , J. Wang , C. Ji , J. Chen , W. Zhao , J. Fan , W. Liu , W. Cai , J. Nanobiotechnology 2020, 18, 105. 32711535 10.1186/s12951-020-00665-8 PMC7382861

[97] K.‐L. Zhou , Y.‐F. Zhou , K. Wu , N.‐F. Tian , Y.‐S. Wu , Y.‐L. Wang , D.‐H. Chen , B. Zhou , X.‐Y. Wang , H.‐Z. Xu , X.‐L. Zhang , Sci. Rep. 2015, 5, 17130. 26597839 10.1038/srep17130 PMC4657088

[98] J. Li , Q. Wang , H. Cai , Z. He , H. Wang , J. Chen , Z. Zheng , J. Yin , Z. Liao , H. Xu , J. Xiao , F. Gong , J. Cell. Mol. Med. 2018, 22, 2727. 29512938 10.1111/jcmm.13566 PMC5908106

[99] C. T. Chu , C. T. Chu , E. D. Plowey , R. K. Dagda , R. W. Hickey , S. J. Cherra , R. S. B. Clark , Methods Enzymol. 2009, 453, 217. 19216909 10.1016/S0076-6879(08)04011-1 PMC2669321

[100] X. Wang , X. Zhang , F. Wang , L. Pang , Z. Xu , X. Li , J. Wu , Y. Song , X. Zhang , J. Xiao , H. Lin , Y. Liu , Clin. Res. Hepatol. Gastroenterol. 2019, 43, 707. 31029643 10.1016/j.clinre.2019.03.006

