# Supplementary material for: NEMO‐Binding Domain/IKKγ Inhibitory Peptide Alleviates Neuronal Pyroptosis in Spinal Cord Injury by Inhibiting ASMase‐Induced Lysosome Membrane Permeabilization
Source: Adv Sci (Weinh). 2024 Sep 3;11(40):2405759. doi: 10.1002/advs.202405759 (PMC11516130; doi:10.1002/advs.202405759)
Supplement: Supplementary file 1 — Supporting Information [file ADVS-11-2405759-s001.docx]

**Supplementary Information**

**For**

NEMO-binding Domain/ IKKγ Inhibitory Peptide Alleviates Neuronal Pyroptosis in Spinal Cord Injury by Inhibiting ASMase-induced Lysosome Membrane Permeabilization

Yibo Geng et. al

**Figures and figure legends**

Figure S1


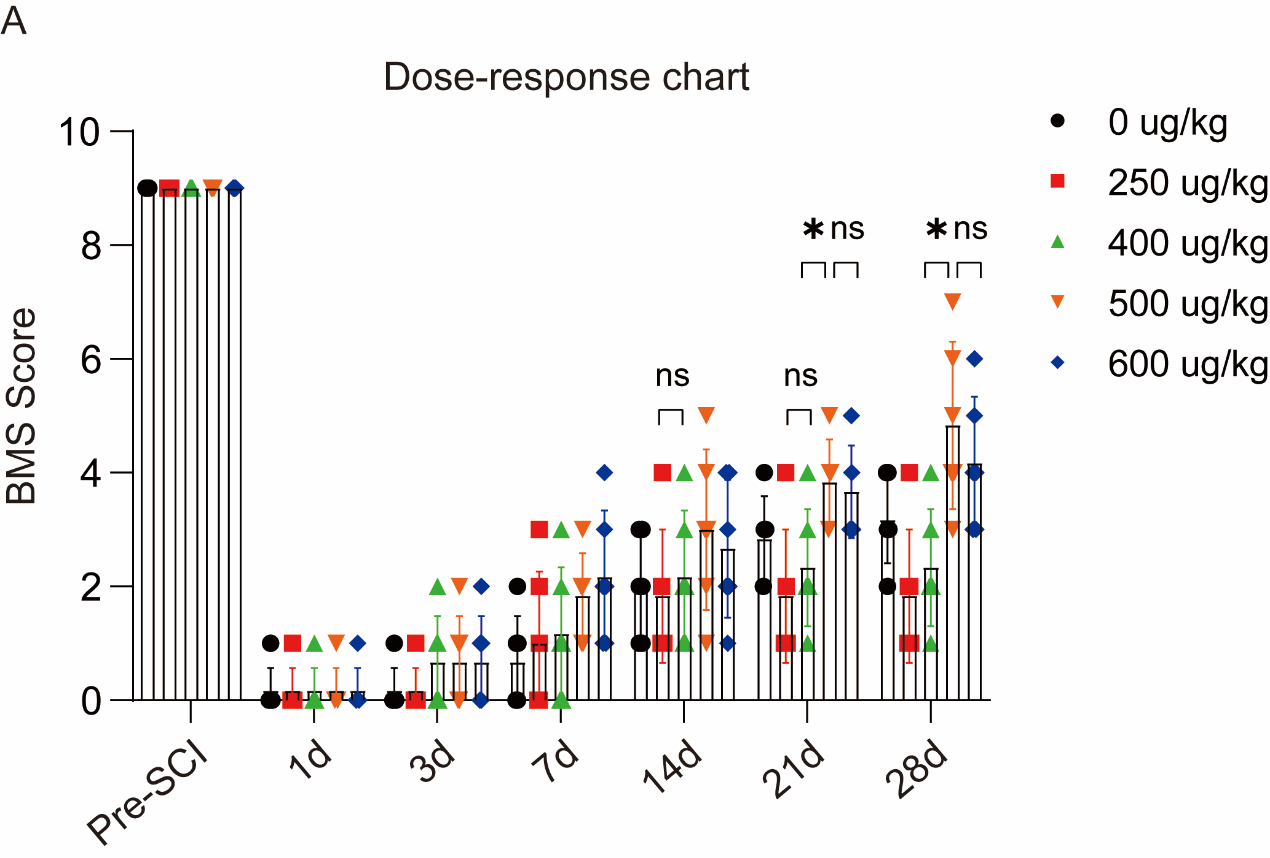


**Fig. S1. Relationship between the various doses of NBD peptide treatment and functional recovery after SCI.** Dose‒response chart showing the optimal dosage of NBD peptide (500 µg/kg) for 28 days after SCI evaluated using the BMS score. The data are presented as the means ± SEMs (n = 6 mice per group); *P < 0.05 indicates significant differences; ns, not significant. Statistical analysis was performed using two-way ANOVA followed by Tukey’s multiple comparison test.

Figure S2


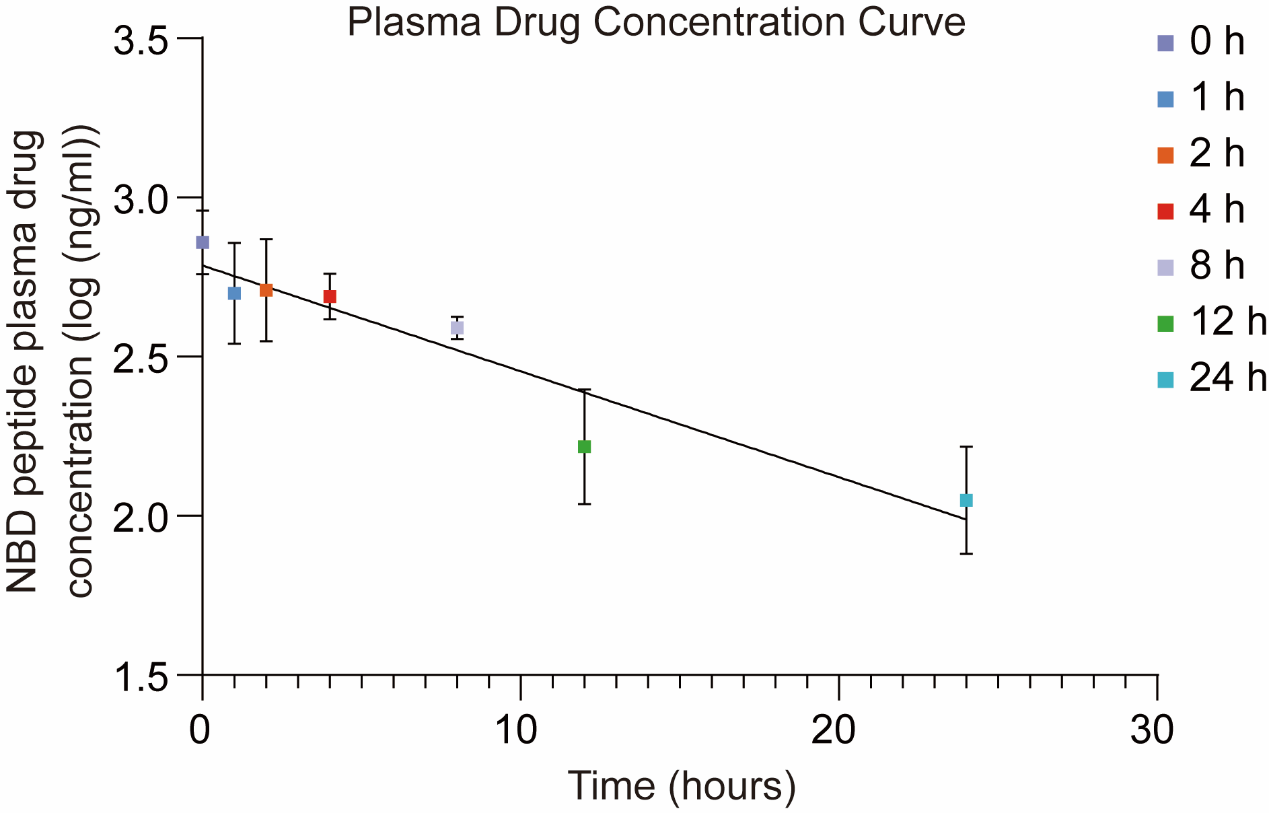


**Fig. S2.** **Time-concentration curves of NBD peptide.** The plasma drug concentration curve shows the plasma concentration of NBD peptide in mice at different time points (0 h, 1 h, 2 h, 4 h, 8 h, 12 h, and 24 h) after the administration of a dose of 500 μg/kg. According to the one-compartment theory, the half-life of NBD peptide was calculated as 20.25 hours. The data are presented as the means ± SEMs (n = 3 mice per group).

Figure S3


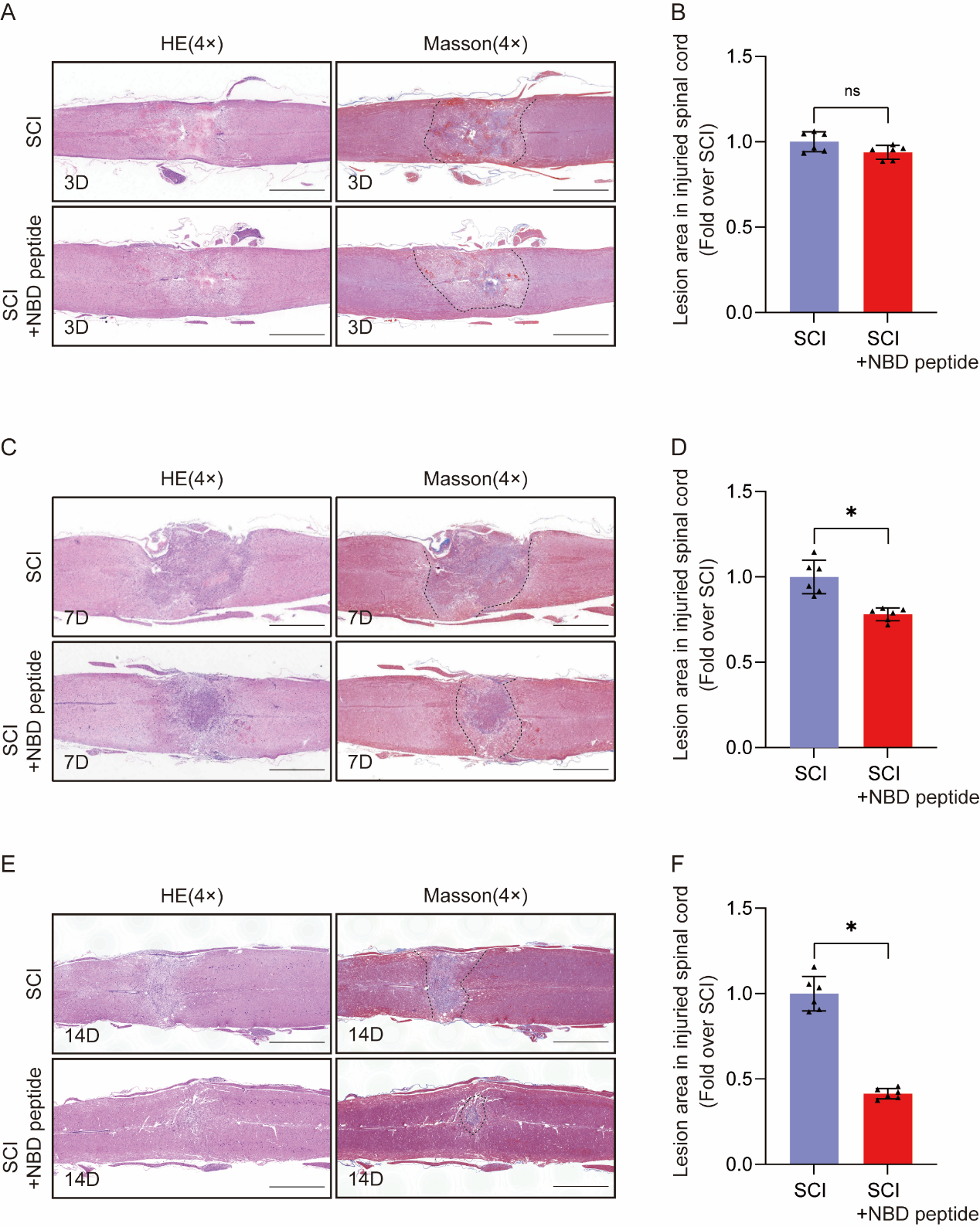


**Fig. S3. NBD peptide promoted an improvement in the histological morphology of the spinal cord after SCI.** (A-B) Longitudinal spinal cord sections from the groups were examined at 3 dpi via HE and Masson staining (scale bar = 1000 μm). Quantitative investigations of Masson-positive lesions within the spinal cords of the different groups. (C-D) Longitudinal sections of spinal cords from the indicated groups were analysed via HE staining and Masson staining on day 7 after SCI (scale bar = 1000 μm). Quantitative analysis of Masson positive lesions in the spinal cords of each group. (E-F) Longitudinal sections of spinal cords from the indicated groups were analysed via HE staining and Masson staining on day 14 after SCI (scale bar = 1000 μm). Quantitative analysis of Masson positive lesions in the spinal cords of each group. The data are presented as the means ± SEMs (n = 6 mice per group); *P < 0.05 indicates significant differences; ns, not significant. Statistical analysis was performed using an unpaired t test.

Figure S4


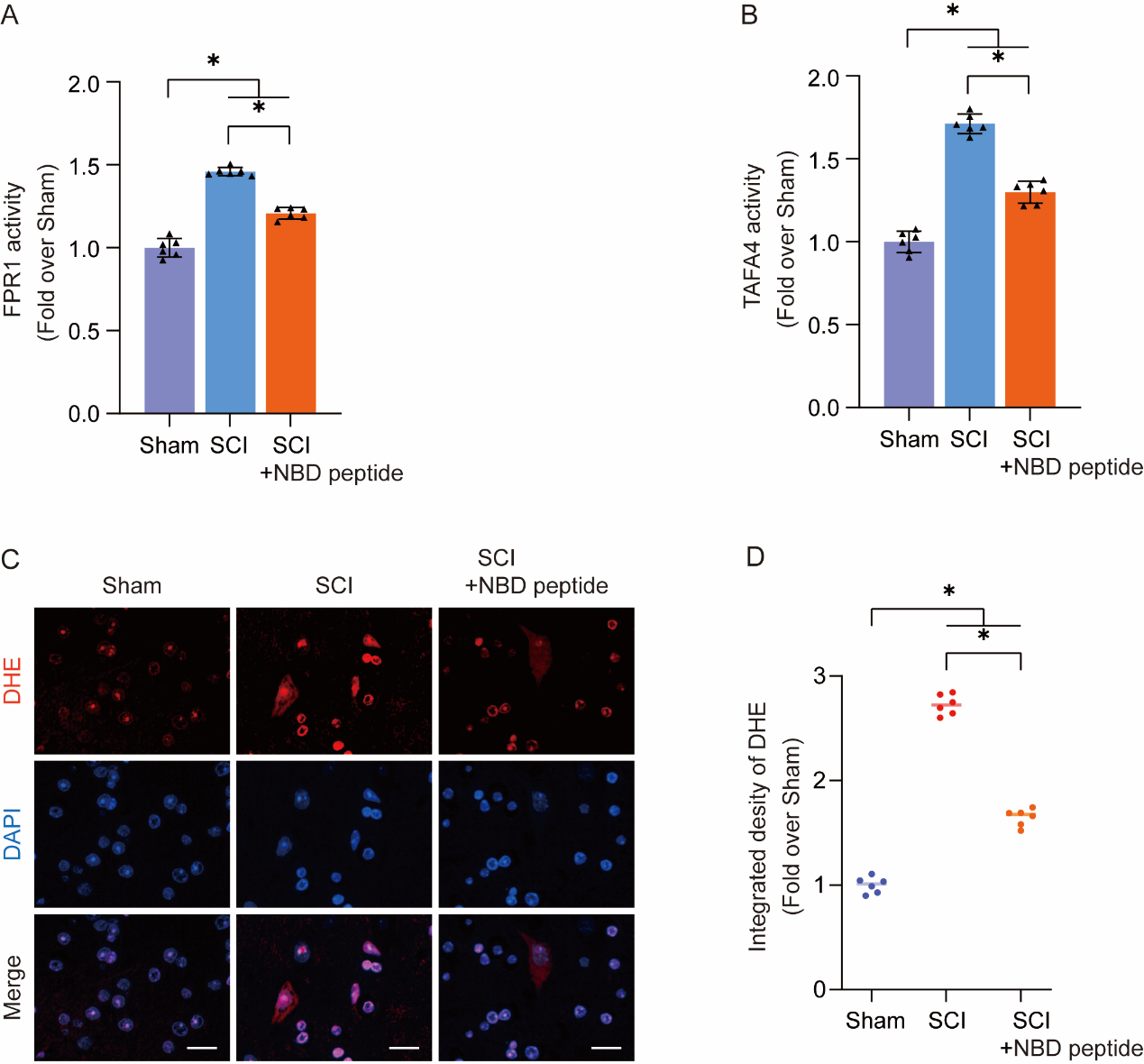


**Fig. S4. NBD peptide inhibits oxidative stress after SCI.** (A-B) ELISA results indicating the levels of FPR1 and TAFA4 activity in the spinal cord of the Sham, SCI, and SCI+NBD peptide groups on the third day after SCI. (C) On day 3 after SCI, frozen spinal cord tissue sections from the three groups were stained with DHE (scale bar = 20 μm). (D) Quantification of DHE staining in the three groups. The data are presented as the means ± SEMs (n = 6 mice per group); *P < 0.05 indicates significant differences; ns, not significant. Statistical analysis was performed using two-way ANOVA followed by Tukey’s multiple comparison test.

Figure S5


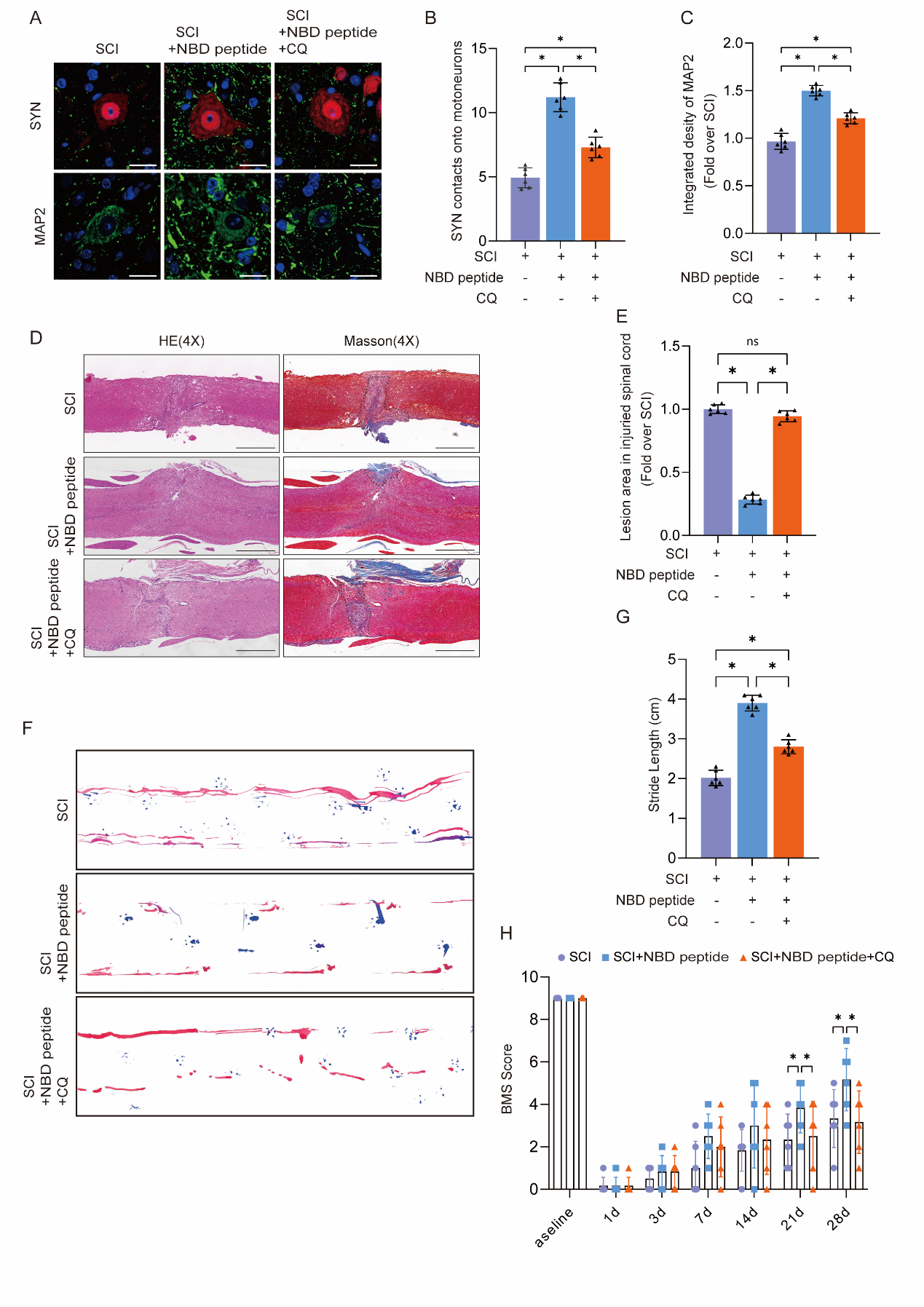


**Fig. S5. Inhibiting autophagy with CQ abrogates NBD peptide-mediated functional recovery following SCI.** (A) Images of spinal cord cross-sections from the corresponding groups subjected to staining for MAP2 (green) and SYN (green)/NeuN (red). (scale bar = 20 μm). (B-C) On day 28 after SCI, the optical density of MAP2 and the number of motor neuron-contacting synapses in the different groups were quantitatively analysed. (D) Longitudinal spinal cord sections obtained at 28 dpi after SCI were stained with HE and Masson staining (scale bar =1000 μm). (E) The lesion area in the injured spinal cord was measured using Masson staining. (F) On day 28 after SCI, images of mouse footprints were captured. Blue: forepaw print; Red: hindpaw print. (G) Analysis of the mouse stride length (cm) at 28 dpi. (H) The BMS scores of the mice in each group were recorded on days 1, 3, 7, 14, 21, and 28 following SCI. The data are presented as the means ± SEMs (n = 6 mice per group); *P < 0.05 indicates significant differences; ns, not significant. Statistical analysis was performed using two-way ANOVA followed by Tukey’s multiple comparison test.

Figure S6


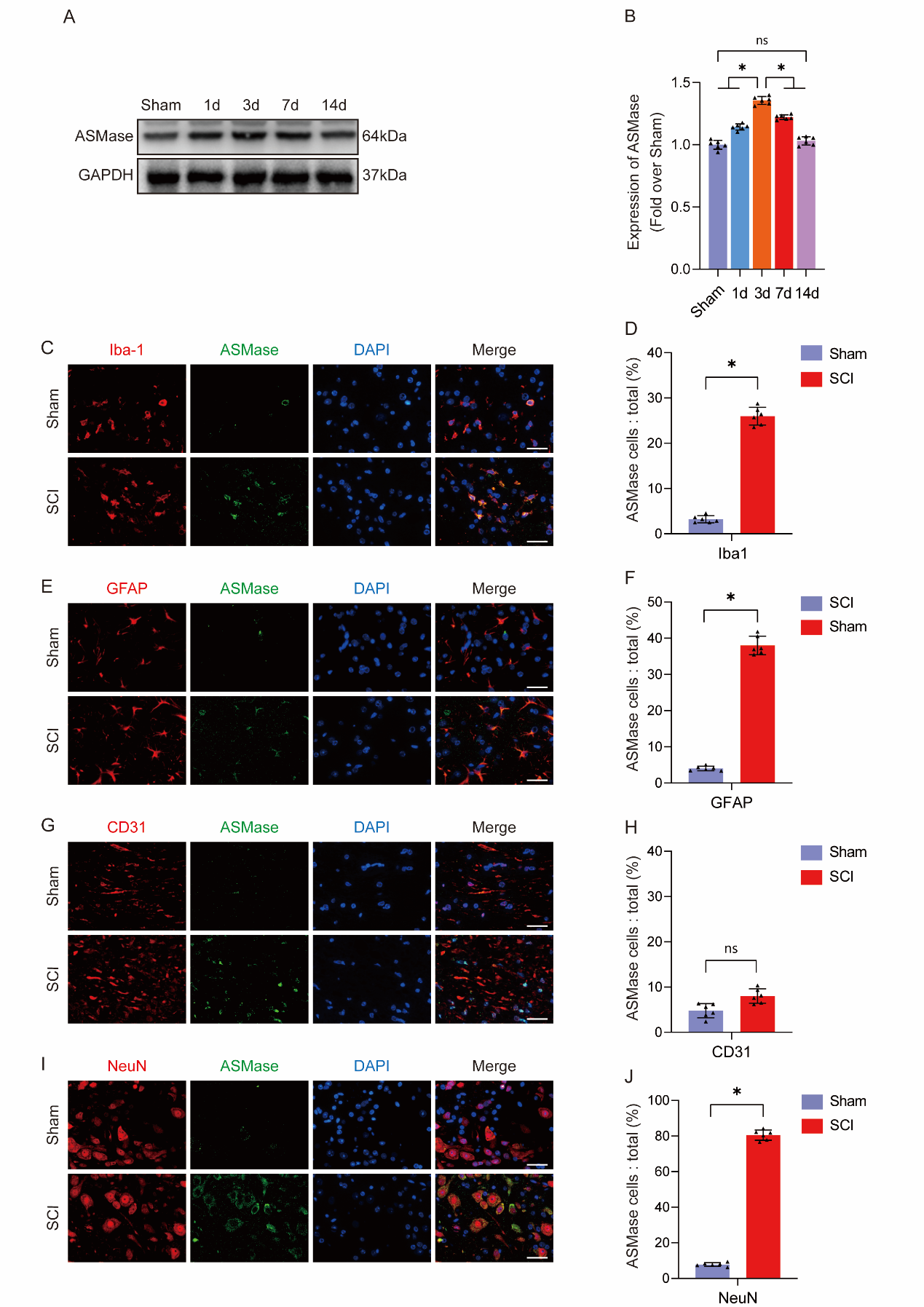


**Fig. S6. The expression levels of ASMase are increased in different cells following SCI.** (A) The level of the ASMase protein was analysed by WB on days 1, 3, 7, and 14 after SCI. GAPDH was used as a loading control. (B) Quantification of the Western blot results for the levels of proteins related to ASMase. (C-D) Typical images of immunofluorescence staining for ASMase and microglial colocalization in the spinal cords of the Sham and SCI groups. The ratio of ASMase-positive cells to total cells is shown on the right (scale bar = 100 μm). (E-F) Typical images of immunofluorescence staining for ASMase and astrocyte colocalization in the spinal cords of the two groups. The ratio of ASMase-positive cells to total cells is shown on the right (scale bar = 100 μm). (G-H) Typical images of immunofluorescence staining for ASMase and endothelial cell colocalization in the spinal cords of the two groups. The ratio of ASMase-positive cells to total cells is shown on the right (scale bar = 100 μm). (I-J) Typical images of immunofluorescence staining for ASMase and neuronal colocalization in the spinal cords of the two groups. The ratio of ASMase-positive cells to total cells is shown on the right (scale bar = 100 μm). The data are presented as the means ± SEMs (n = 6 mice per group); *P < 0.05 indicates significant differences; ns, not significant. For the data in Fig. S6B, the statistical analysis was performed using one-way ANOVA followed by the LSD post hoc analysis (equal variances of the groups) or Dunnett's T3 method (unequal variances of the groups). For the data in Fig. S6D, F, H and J, the statistical analysis was performed using unpaired t tests.

Figure S7


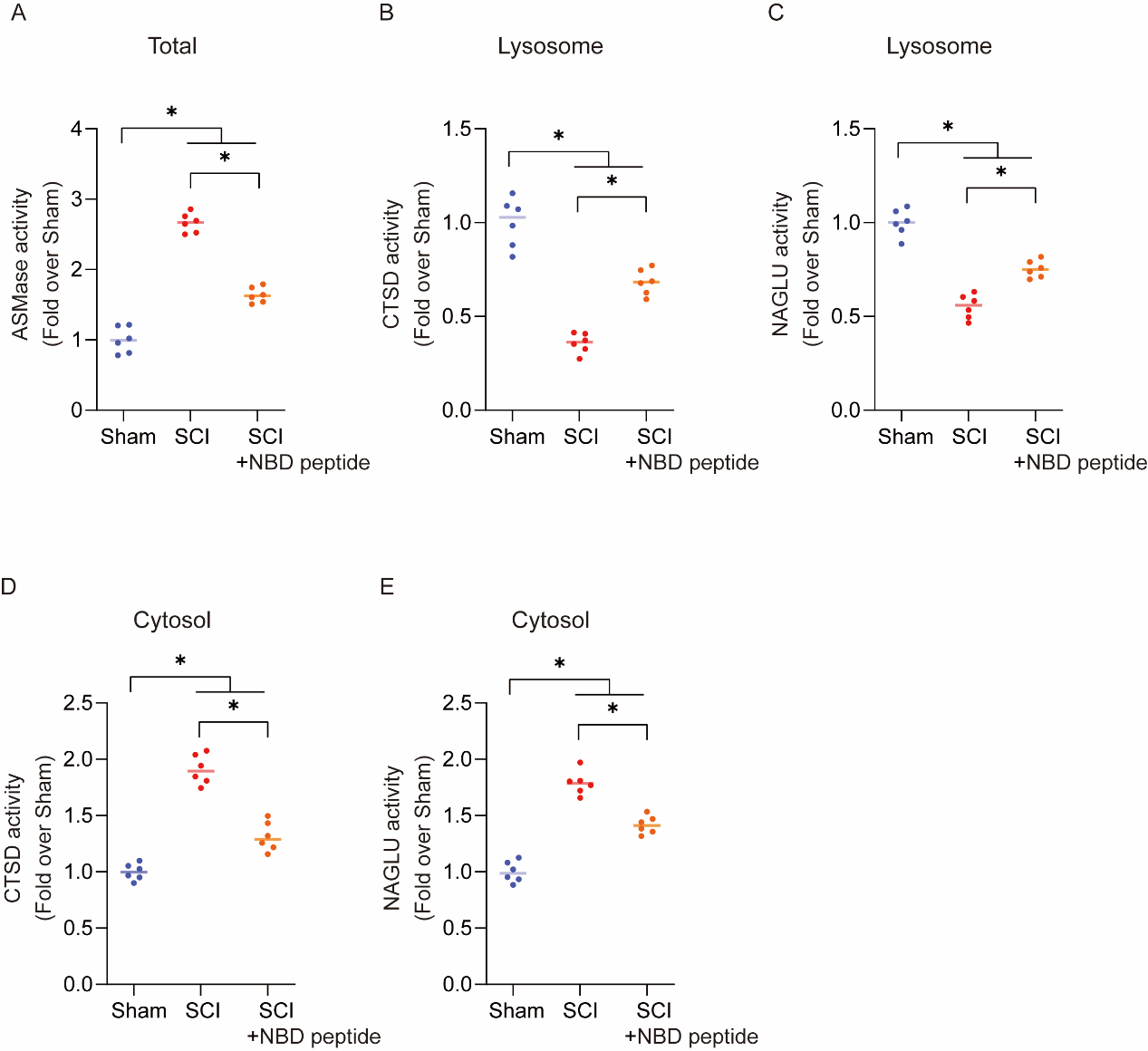


**Fig. S7. NBD peptide attenuates LMP and inhibits the activity of ASMase after SCI.** (A) ELISA results indicating the level of ASMase activity in the spinal cord of the Sham, SCI, and SCI+NBD peptide groups on the third day after surgery. (B-E) ELISA was used to determine the activity of the lysosomal enzymes CTSD and NAGLU in both the lysosomal (B, C) and cytosolic (D, E) fractions obtained from the spinal cords of the Sham, SCI, and SCI+NBD peptide groups on day 3 postsurgery. The data are presented as the means ± SEMs (n = 6 mice per group); *P < 0.05 indicates significant differences; ns, not significant. Statistical analysis was performed using two-way ANOVA followed by Tukey’s multiple comparison test.

Figure S8


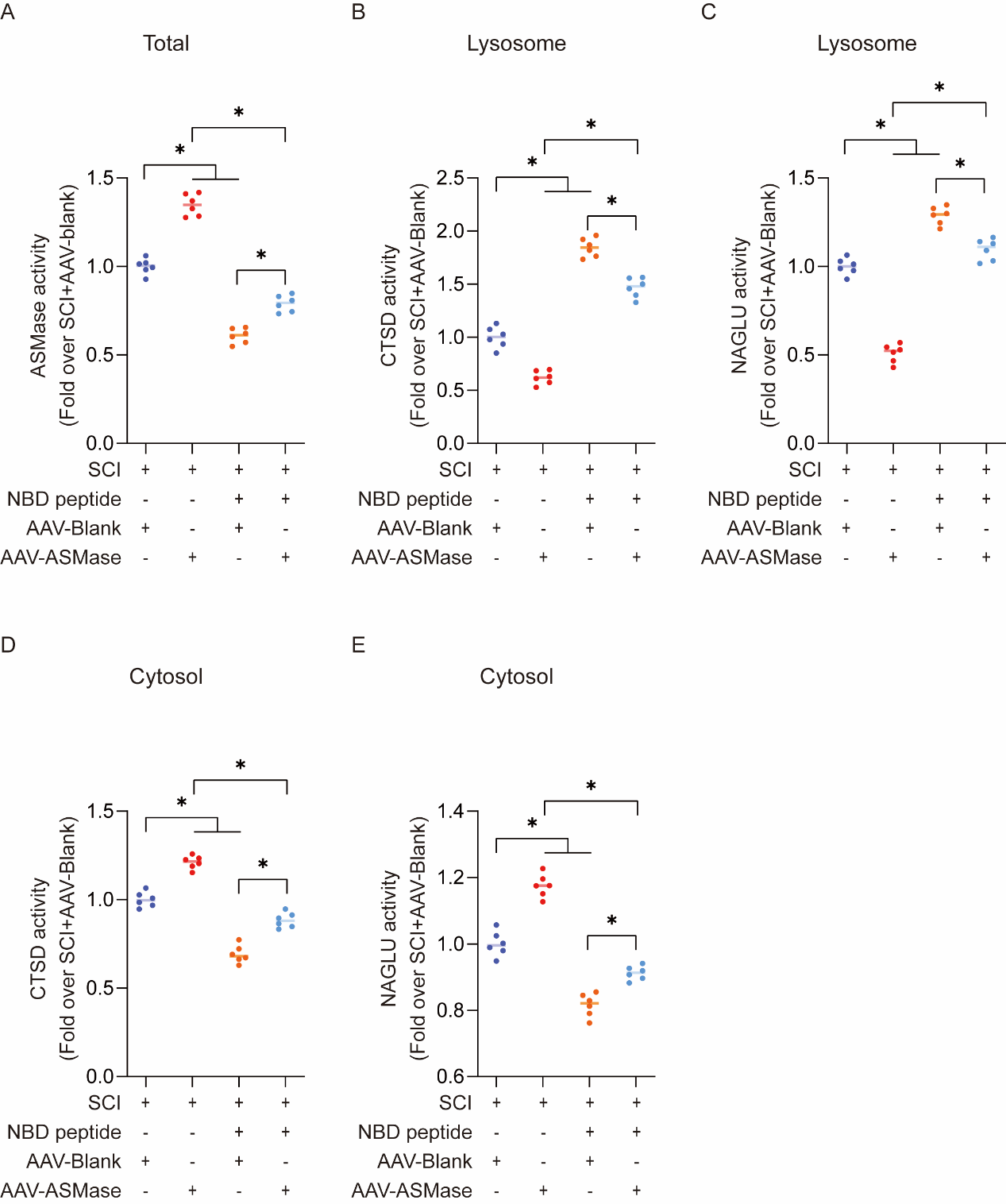


**Fig. S8. NBD peptide attenuates LMP by inhibiting ASMase in SCI model mice.** (A) ELISA results indicating the level of ASMase activity in the spinal cord of the SCI+AAV-Blank, SCI+AAV-ASMase, SCI+NBD peptide+AAV-Blank, and SCI+NBD peptide+AAV-ASMase groups on the third day after surgery. (B-E) ELISA was used to determine the activity of the lysosomal enzymes CTSD and NAGLU in both the lysosomal (B, C) and cytosolic (D, E) fractions obtained from the spinal cords of the SCI+AAV-Blank, SCI+AAV-ASMase, SCI+NBD peptide+AAV-Blank, and SCI+NBD peptide+AAV-ASMase groups on day 3 postsurgery. The data are presented as the means ± SEMs (n = 6 mice per group); *P < 0.05 indicates significant differences; ns, not significant. Statistical analysis was performed using two-way ANOVA followed by Tukey’s multiple comparison test.

Figure S9


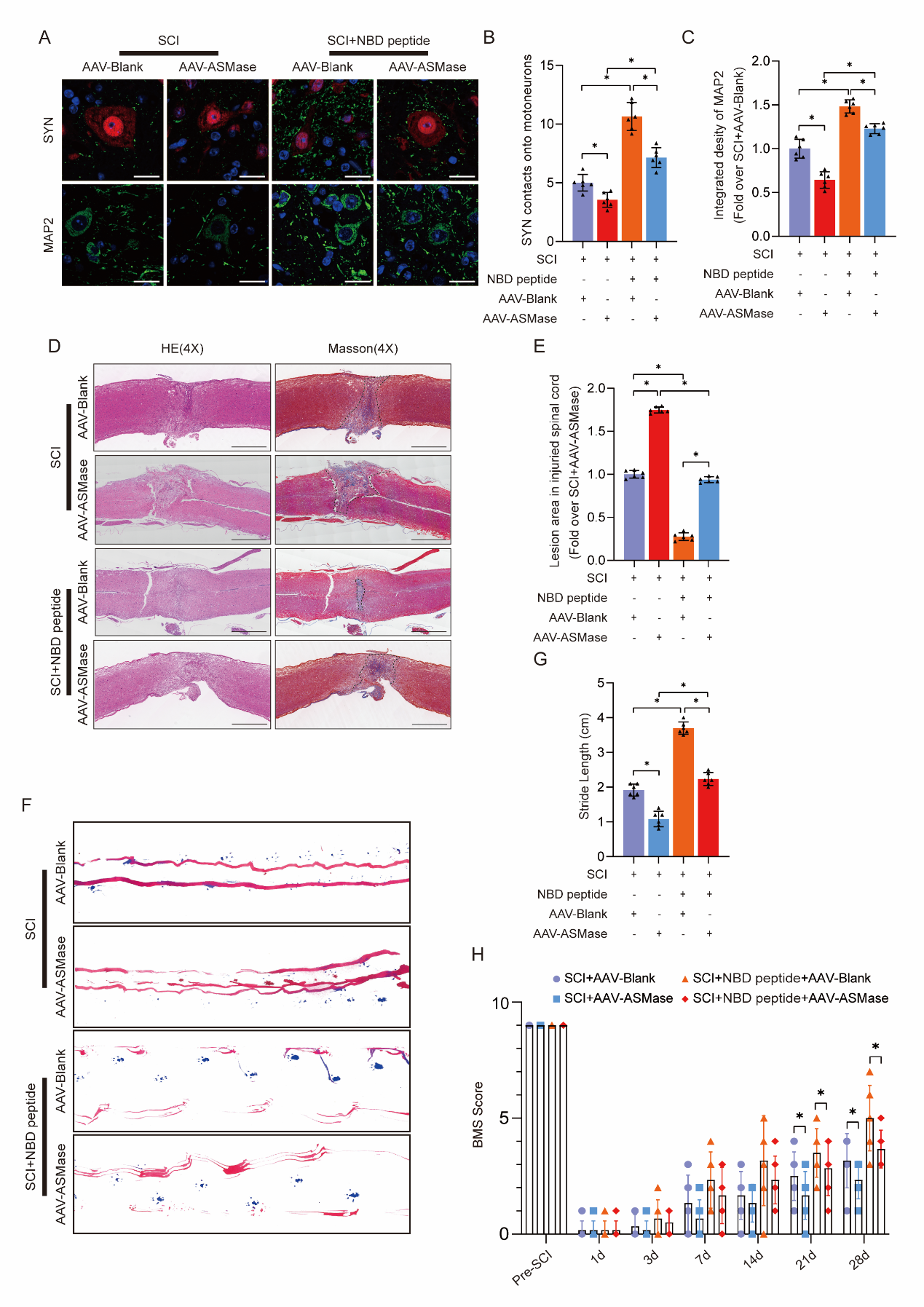


**Fig. S9. NBD peptide facilitates functional recovery in SCI mice by inhibiting ASMase activity.** (A) Images of MAP2 (green)- and SYN (green)/NeuN (red)-stained spinal cord sections from the corresponding groups. (scale bar = 20 μm). (B-C) On day 28 after SCI, the optical density of MAP2 and the number of motor neuron-contacting synapses in the different groups were quantitatively analysed. (D) Longitudinal spinal cord sections obtained at 28 dpi after SCI were stained with HE and Masson staining (scale bar = 1000 μm). (E) The lesion area in the injured spinal cord was measured using Masson staining. (F) On day 28 after SCI, images of mouse footprints were captured. Blue: forepaw print; Red: hindpaw print. (G) Analysis of the mouse stride length (cm) at 28 dpi. (H) The BMS scores of the mice in each group were recorded on days 1, 3, 7, 14, 21, and 28 following SCI. The data are presented as the means ± SEMs (n = 6 mice per group); *P < 0.05 indicates significant differences; ns, not significant. Statistical analysis was performed using two-way ANOVA followed by Tukey’s multiple comparison test.

Figure S10


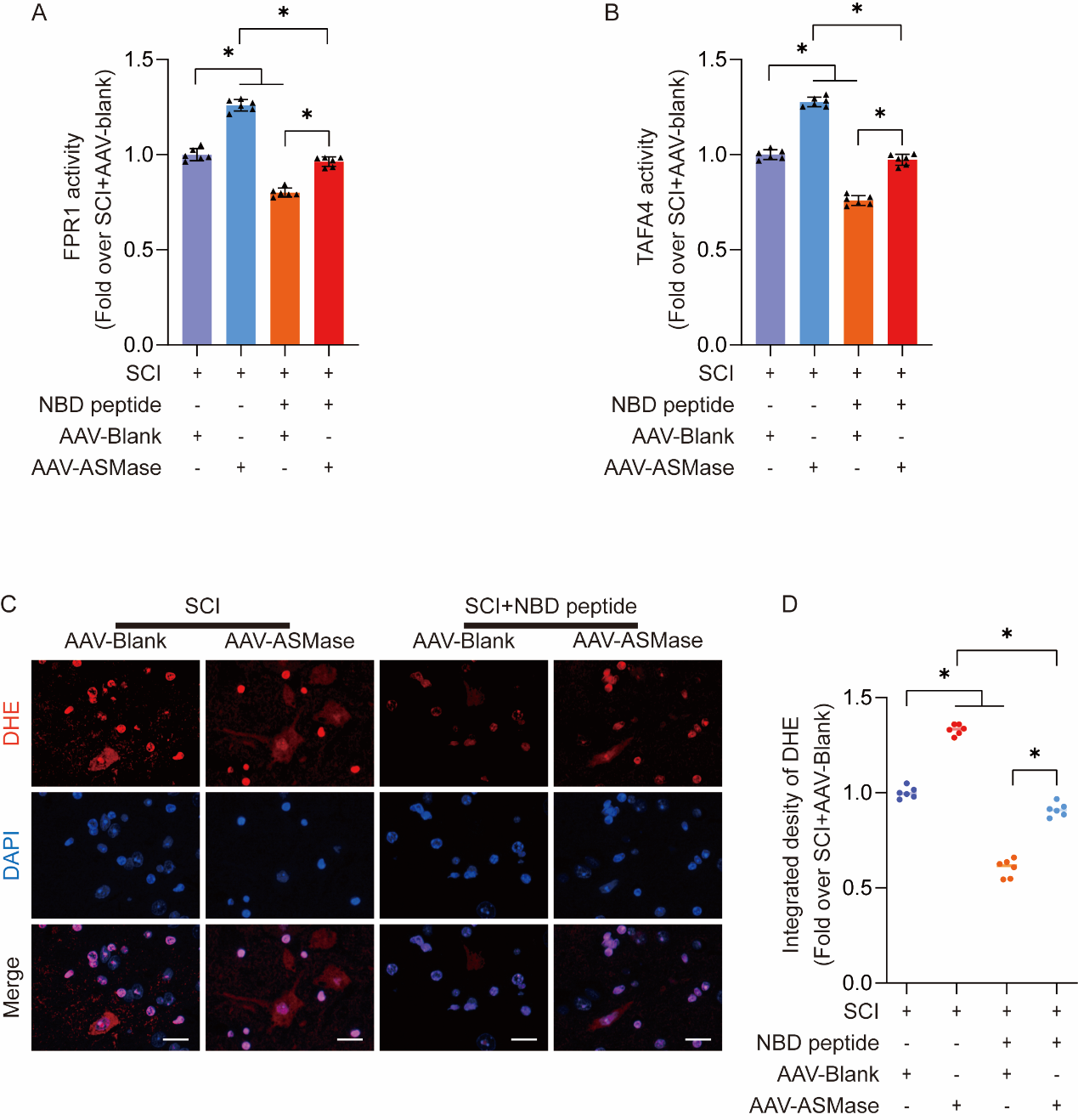


**Fig. S10. NBD peptide inhibits oxidative stress after SCI by suppressing ASMase activity.** (A-B) ELISA results indicating the levels of FPR1 and TAFA4 activity in the spinal cord of the SCI+AAV-Blank, SCI+AAV-ASMase, SCI+NBD peptide+AAV-Blank, and SCI+NBD peptide+AAV-ASMase groups on the third day after surgery. (C) On day 3 after SCI, frozen spinal cord tissue sections from the 4 groups were stained with DHE (scale bar = 20 μm). (D) Quantification of DHE staining in the 4 groups. The data are presented as the means ± SEMs (n = 6 mice per group); *P < 0.05 indicates significant differences; ns, not significant. Statistical analysis was performed using two-way ANOVA followed by Tukey’s multiple comparison test.

Figure S11

**
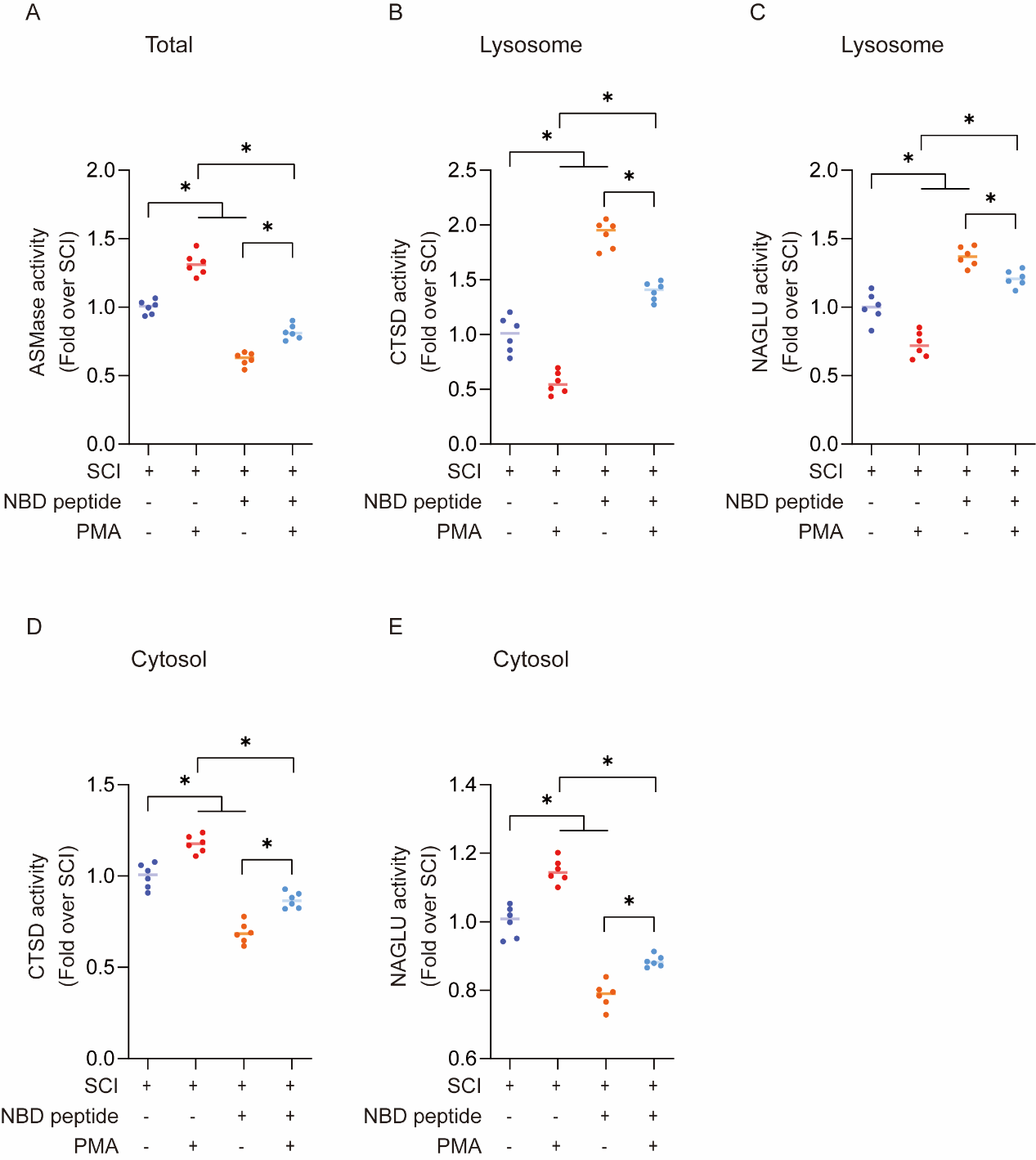
**

**Fig. S11. NBD peptide attenuates LMP by inhibiting the NF-κB/p38-MAPK/ElK-1/Egr-1 signaling pathway.** (A) The ELISA results indicating the level of ASMase activity in the spinal cords of the SCI, SCI+PMA, SCI+NBD peptide, and SCI+NBD peptide+PMA groups on the third day after surgery. (B-E) ELISA was used to determine the activity of the lysosomal enzymes CTSD and NAGLU in both the lysosomal (B, C) and cytosolic (D, E) fractions obtained from the spinal cords of the SCI, SCI+NBD peptide, and SCI+NBD peptide+PMA groups on day 3 postsurgery. The data are presented as the means ± SEMs (n = 6 mice per group); *P < 0.05 indicates significant differences; ns, not significant. Statistical analysis was performed using two-way ANOVA followed by Tukey’s multiple comparison test.

Figure S12


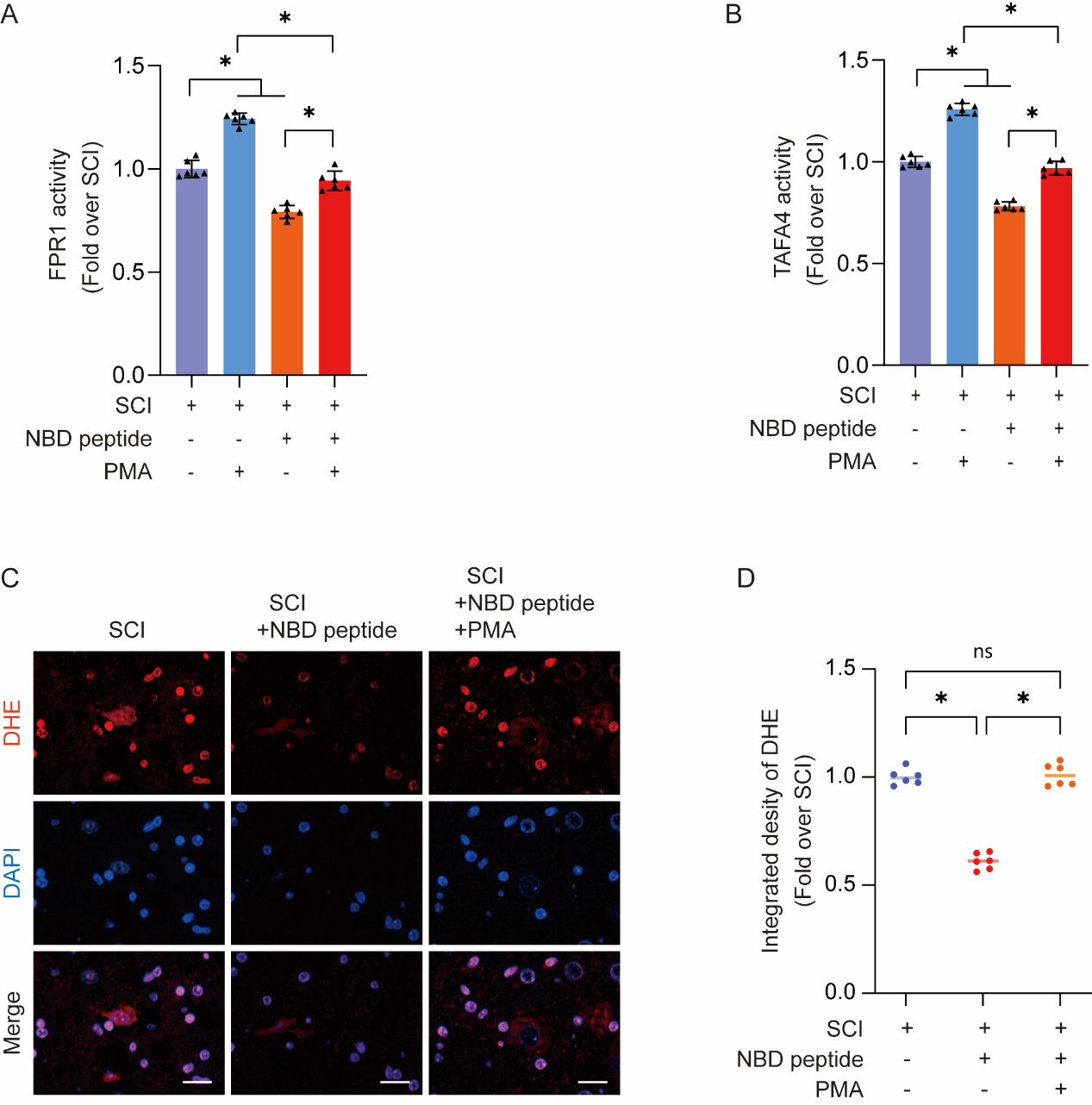


**Fig. S12. NBD peptide inhibits oxidative stress by inhibiting NF-κB.** (A-B) ELISA results indicating the levels of FPR1 and TAFA4 activities in the spinal cords of the SCI, SCI+PMA, SCI+NBD peptide, and SCI+NBD peptide+PMA groups on the third day after surgery. (C) On day 3 after SCI, frozen spinal cord tissue sections from the 4 groups were stained with DHE (scale bar = 20 μm). (D) Quantification of DHE staining in the 4 groups. The data are presented as the means ± SEMs (n = 6 mice per group); *P < 0.05 indicates significant differences; ns, not significant. Statistical analysis was performed using two-way ANOVA followed by Tukey’s multiple comparison test.
